# Folding and Evolution of a Repeat Protein on the Ribosome

**DOI:** 10.3389/fmolb.2022.851038

**Published:** 2022-05-30

**Authors:** José Alberto León-González, Perline Flatet, María Soledad Juárez-Ramírez, José Arcadio Farías-Rico

**Affiliations:** ^1^ Synthetic Biology Program, Center for Genome Sciences, National Autonomous University of Mexico, Cuernavaca, Mexico; ^2^ Department of Biochemistry and Biophysics, Stockholm University, Stockholm, Sweden

**Keywords:** evolution, ribosome, protein, folding, cotranslational

## Abstract

Life on earth is the result of the work of proteins, the cellular nanomachines that fold into elaborated 3D structures to perform their functions. The ribosome synthesizes all the proteins of the biosphere, and many of them begin to fold during translation in a process known as cotranslational folding. In this work we discuss current advances of this field and provide computational and experimental data that highlight the role of ribosome in the evolution of protein structures. First, we used the sequence of the Ankyrin domain from the *Drosophila* Notch receptor to launch a deep sequence-based search. With this strategy, we found a conserved 33-residue motif shared by different protein folds. Then, to see how the vectorial addition of the motif would generate a full structure we measured the folding on the ribosome of the Ankyrin repeat protein. Not only the on-ribosome folding data is in full agreement with classical *in vitro* biophysical measurements but also it provides experimental evidence on how folded proteins could have evolved by duplication and fusion of smaller fragments in the RNA world. Overall, we discuss how the ribosomal exit tunnel could be conceptualized as an active site that is under evolutionary pressure to influence protein folding.

## Introduction

During the past 50 years we have learned much about how purified proteins fold in diluted buffer conditions (aka *in vitro* folding). Today for example, there are available powerful computational models that can predict with great accuracy the folded structure of small single-domain proteins with two-state folding kinetics behavior. However, proteins do not fold in isolation in the cell; for instance, many proteins from the *E. coli* proteome are not intrinsically refoldable under physiological conditions (To et al., 2021). They seem to require another cellular element to reach their native state. To bridge the gap between classical *in vitro* folding studies and more biologically relevant conditions many efforts have been made to increase our understanding on how proteins fold in the crowded cellular environment. The ribosome is the first proteostasis hotspot of the cell, representing a good place where folding could start being regulated. Early work demonstrated secondary structure formation deep in the ribosomal exit tunnel (Lu and Deutsch, 2005); more recent studies have shown folding of small full domains (Nilsson et al., 2015). And lately, we have witnessed the image of a fully folded Ig domain in the vestibule of the ribosome (Tian et al., 2018).

Other research groups using elegant biochemical methods have demonstrated that average domain-size proteins (>100 aa) folding up to 55 residues away from the peptidyl transferase center (PTC) experience a decrease in thermodynamic stability (Samelson et al., 2016). Interestingly, a small zinc finger domain is more stable and folds faster deep in the tunnel (at 26 residues away from the PTC) than right at the vestibule of the ribosomal tunnel (at 34 residues from the PTC) (Wruck et al., 2021). The authors argue that electrostatic interactions established between the walls deep in the tunnel and the small protein are the source of this increase. Alternatively, the same kind of interactions established by a protein folding outside of the ribosome are characterized as a competition between folding and binding (Cassaignau et al., 2021). These authors conceptualize the ribosome as a holdase that prevents aggregation during cotranslational folding. With no doubt, the holdase function of the ribosome must be of particular importance during the folding of multidomain proteins. For example, single molecule experiments have demonstrated that interactions with the ribosome compete with interdomain misfolding providing another layer for proteostasis regulation (Liu et al., 2019).

Evolution shapes every aspect of the biological world; for instance, in the case of cellular proteostasis, which relies on the precise coordination of translation and folding (Waudby et al., 2019), it has been proposed an evolutionary selection for clusters of rare codons (Jacobs and Shakhnovich, 2017). In this work it is argued that conservation of rare codons clusters is coincident with the prediction of folding intermediates in the nascent chains. Other works have also suggested a match between conserved clusters of rare or optimal codons (Pechmann and Frydman, 2013; Chaney et al., 2017) and secondary or super-secondary structures, such as the βαβ motif, that are foldable within the exit tunnel. More recently, these foldable units have been experimentally mapped onto the full folding trajectory on the ribosome of a small helical domain (Liutkute et al., 2020). The authors of this work leveraged on the development of a novel assay based on arrest peptides to measure the force that the nascent chain exerts on the ribosome (Force Profile Assay, or FPA) (Cymer et al., 2015). Remarkably, they produced a detailed force profile (almost at codon resolution) to uncover the folding trajectory of the HemK. The mechanism described by the authors was characterized as the individual folding of cooperative units or *foldons* that initially fold fast and later compact into the full native state.

An initial definition of the basic units of globular proteins was already made almost 30 years ago by experimentally analyzing the folding of the bovine pancreatic trypsin Inhibitor (Ittah and Haas, 1995). These units were defined as the longest loops that are held together by non-local interactions under folding conditions. Around the same time, another laboratory used hydrogen exchange to show that cytochrome C folds via cooperative units or foldons of 15–27 residues in size (Bai et al., 1995). They point out that within the context of the Levinthal paradox, it would be easier to fold a few cooperative 15–27 residue segments than a whole 100 residue molecule. Later, by an exhaustive examination of the curvature of protein backbones these elements (named as foldons) were generalized as universal basic units of protein structures (Berezovsky et al., 2000). The ends of the foldons were defined as chain-to chain contacts with Cα to Cα distances smaller than 10 Å.

Interestingly, the ends of the loops coincided well with peaks in hydrophobicity of many protein folds, therefore its definition was later refined to include hydrophobic interactions (Berezovsky et al., 2001). This correlation demonstrates not only the importance of the hydrophobic nuclei in protein folding but also the role of the foldons of 20–50 residues as primary building blocks. If cooperative folding units or foldons are the works of evolution, one must be able to find traces of its conservation not only at the codon level but also at the protein structure level. The use of sequence conservation to detect residues important for folding dates back to the 1990s (Shakhnovich et al., 1996). This seminal work set the stage for a heated debate on whether it was possible to detect the folding nucleus by relying on conservation of non-functional residues. Later, by grouping amino acids according to their physical chemistry properties and applying an adequate normalization, it was demonstrated that the folding nucleus was indeed more conserved than the rest of the protein (Mirny and Shakhnovich, 2001).

This finding was questioned by other groups (Larson et al., 2002; Tseng and Liang, 2004) arguing that the folding process was mainly governed by protein topology and both poorly and highly conserved residues are similarly expected to participate in the protein-folding nucleus. At the end of the debate in 2006 it was concluded that the methods to detect conservation were not sensitive enough to extract this type of signal yet (Shakhnovich, 2006). More recently however, one study analyzing the highly populated TIM barrel fold found evidence of conservation of the refolding mechanism in a family of close homologs (Carstensen, Sperl et al., 2012). And nowadays, it was possible to detect conservation in the folding mechanism of two TIM-barrel fold proteins from different kingdoms (Jain, Muneeruddin et al., 2021). These observations are believed to indicate a conservation of folding mechanisms since the Last Universal Common Ancestor (LUCA).

It is not the first time that subdomain-sized foldons or fragments are invoked to explain features of the structural proteomes. Several laboratories have applied state-of-the-art tools for homology detection, structural comparisons and bioinformatic pipelines (Cheng et al., 2014; Alva et al., 2015; Ferruz et al., 2020; Kolodny et al., 2021) to describe how nature has tinkered with subdomain-sized fragments to create the vast protein structural diversity that we observe today. Most protein evolution studies based on sequence comparisons, however, have limited their analysis to fully folded protein structures deposited in the PDB (Burley et al., 2021) and have not explored kinetic intermediates. Perhaps because the study of shorter-lived intermediates and folding pathways is a very difficult task (Wensley et al., 2010).

With the development of more sensitive methods for homology detection (Remmert et al., 2011), we were able to find evidences of evolutionary relationships between the Flavodoxin-like and the TIM-barrel folds (Farias-Rico et al., 2014). Interestingly, the connection between the two folds was established by a foldon-size unit. Similar studies discovered a likely evolutionary connection between two ancestral protein architectures: the P-loop NTPases and the Rossman fold (Longo et al., 2020). The connecting theme was composed by a β-(phosphate binding loop)-αβ super-secondary structure of around 30 residues.

Other authors (Alva et al., 2015) have found a set of 40 ancient fragments that link many folds and are also foldon-size units (30–40 aa). These discoveries are remarkable because the size of these fragments perfectly correspond to the size of the zinc finger domain adr1 (29 aa) that is folded deep in the ribosomal tunnel (Nilsson et al., 2015). And what is more, the sub-domain sized fragments have been found in very distant pairs of proteins that are not considered homologous; therefore these recurring themes could be traced over evolutionary time to (Bowman et al., 2020) the origin of the ribosome itself or even to a pre-ribosomal world lacking modern protein translation (Caetano-Anolles et al., 2009). Repetitive themes that homo-oligomerize to form soluble mini proteins could have characterized this early peptide-polynucleotide stage of protein fold evolution (Fried et al., 2022).

Many authors have highlighted the advantages of studying the folding pathways of homologous proteins (Nickson and Clarke, 2010), but it is still a matter of debate if a kinetic intermediate could be detected by sequence comparisons. Part of the complication is due to the fact that the folding nucleus of globular domains can migrate to different parts of the protein upon events such as circular permutations (Haglund et al., 2008). In this case, thermodynamically stable structural motifs might not correspond to kinetic intermediates in the folding pathway. We argue however, that the situation could be different if we analyze the folding of a repeat protein. On the contrary to classical globular domains, such as hemoglobin or DHFR, elongated repetitive proteins display low contact order and identical units that fold individually to finally add up to the native structure. In this case, the likelihood that the repetitive motif corresponds to a foldon should be much higher.

After considering the conservationism of conservationism (CoC) approach, which is a strategy that looks for conservation of residue positions in folds across protein families to detect the folding nuclei (Donald et al., 2005), we selected a repetitive protein to query databases in a sequence-based search for foldons. We aimed to find shared motifs in proteins belonging to at least different superfamilies, if not better different folds. In fact, several approaches (Cheng et al., 2014; Kolodny et al., 2021) already have looked for such bridging themes in different folds and hypothesized their role in the evolution of the folded proteins. Our hypothesis is that to study how foldons could be recycled during protein evolution, an obvious candidate may be a repetitive protein. We reasoned that a folding unit or foldon that has not undergone plastic adaptation to a new structural environment could be present in a repeat protein, such as the Ankyrin Domain of the *Drosophila* Notch Receptor.

**FIGURE 1:**
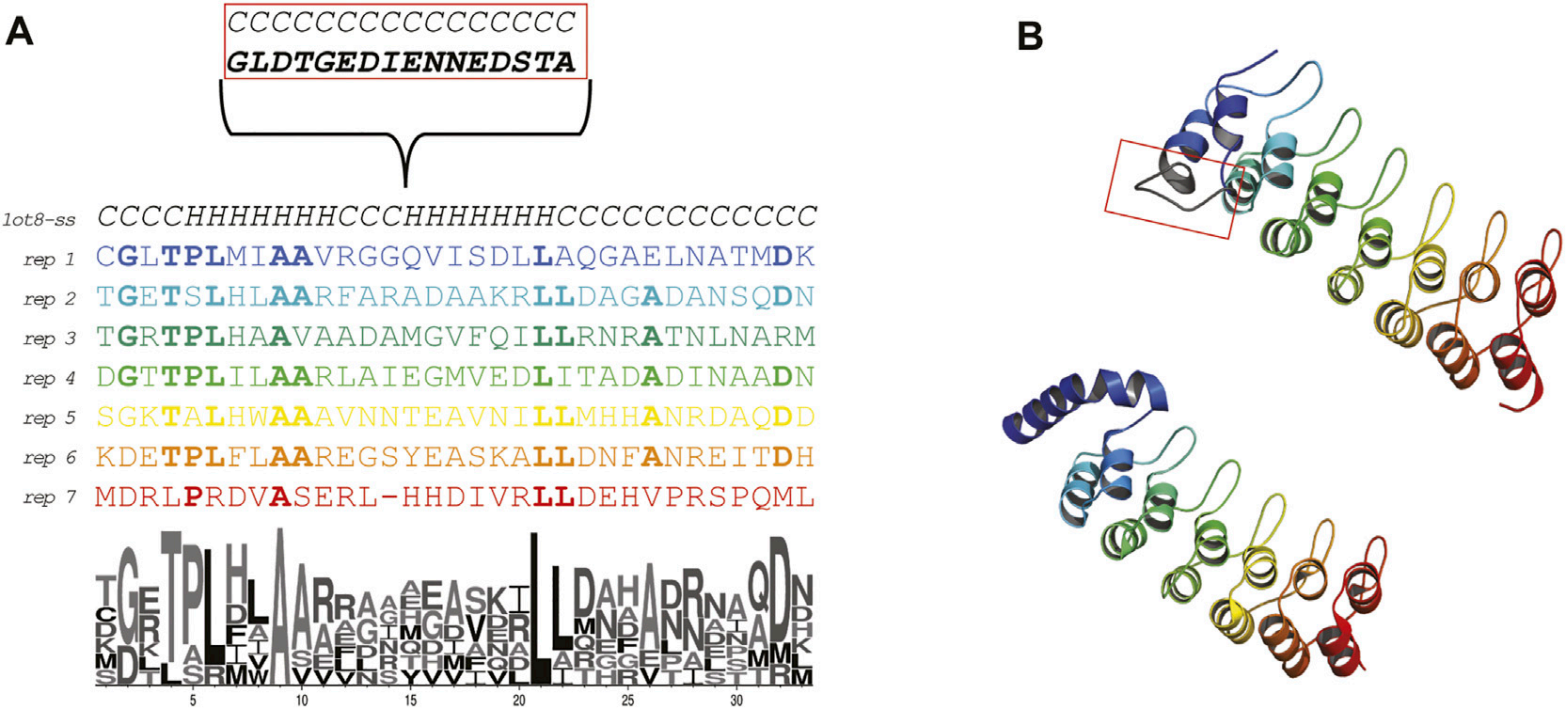
Sequence and structure of the Ankyrin Domain of the *Drosophila* Notch Receptor (Uniprot P07207; PDB: 1ot8). **(A)** Alignment of the repeats forming the Ankyrin repeat protein studied in this work. The secondary structure content is indicated. The first repeat displays an insertion that is involved in protein binding (biological native protein function). A LOGO representation is shown to highlight the residue conservation of the repeats. **(B)** X-ray crystal structure colored by repeat (bottom) and *ab initio* model (top). The conformation of the first repeat in the crystal structure seems to be different than the rest of the repeats (probably due to a crystallization artifact, 26 ATOM records are missing in all chains). Due to the relevance of the repeats for this work, we *ab initio* modeled the protein using the Rosetta.

The Ankyrin Domain of the *Drosophila* Notch Receptor is a repeat protein over-represented in protein databases (Li et al., 2006) and it is mainly functioning to facilitate protein-protein interactions in all domains of life (Kumar and Balbach, 2021). It has been widely engineered to perform several antibody-like functions (Mittl et al., 2020). Each repeat (Figure 1, panel A and B) displays a helix-turn-helix conformation and has high sequence conservation among the repeats featuring two alanines important for folding (Bradley and Barrick, 2002). By addition of repeats these proteins have evolved elongated shapes expanding from 3 to 33 repetitions of the motif.

In the structural classification of proteins (SCOP) (Fox et al., 2014) the Notch domain from *Drosophila Melanogaster* (Protein Data Bank id: 1OT8) is classified as belonging to the beta-hairpin-alpha-hairpin repeat fold (SCOP d.211.1) with multiple repeats of the β_2_-α_2_ unit. We searched the SCOP95 database of proteins using the sequence of the Ankyrin repeat protein and found a sequence-based relationship with three different folds. All the folds shared a common 33 aa sequence theme. We then proceeded to experimentally characterize the folding on the ribosome of this ankyrin repeat protein to hypothesized how the fold could have arisen by duplication and fusion of repeats/themes.

## Materials and Methods

### Protein Structure Models

For the structural studies we downloaded the coordinates of the Ankyrin Domain of the *Drosophila* Notch Receptor (PDB: 1ot8) (Zweifel et al., 2003). The deposited structure contains three chains displaying different degrees of flexibility in the N-terminal side. The most complete chain (A) did not display atom records for the first 26 residues. This is caused by a 15 aa residue insertion (Zweifel and Barrick, 2001). To obtain structural information for the missing ATOM records in the N-terminal side of the structure, we performed *ab initio* modelling of the complete structure with Rosetta (Raman et al., 2009) (Figure 1 panel B top model). The insertion was modelled as an extended loop, and the rest of the repeats look similar in structure to the actual coordinates.

### Repeat Alignment and Logo Generation

The repeats were aligned according to (Zweifel and Barrick, 2001) and a logo representation was generated using the web logo server (Crooks et al., 2004).

### Sequence Comparisons by HMM-HMM Profile Alignments

The automatic sequence-based comparisons between the Ankyrin Domain of the *Drosophila* Notch Receptor (SCOP: d.211.1.1) and the rest of the folds were performed with HHsearch (Soding, 2005) implemented in the web server database Fuzzle 2.0 (Ferruz et al., 2021). The search was performed with default parameters; however, the secondary structure alignment was not scored to avoid biases introduced by the secondary structure contents. All probability hits are recorded based on the Bayesian posterior probability score associated with Hhsearch (Supplementary Figure S1, and Supplementary File S2 raw output of *hhsearch*). The alignment performed by HHsearch maximizes the probability that two HMMs will emit the same sequence of residues. The weighting of the amino acids is in function of their representation in proteins; rare residues will contribute more to the total alignment score. The probability reported by HHsearch is based on the real-world score distribution for negative and homologous domain pairs in an all-against-all comparison of the SCOP database (Soding, 2005).

### Enzymes and Chemicals

All enzymes were purchased from Thermo Scientific (Waltham, MA, United States) and New England Biolabs (Ipswich, MA, United States). Oligonucleotides were obtained from T4 OLIGO (Irapuato, Mexico). DNA purification kits were from Qiagen (Hilden, Germany). The *in vitro* translation system (New England Biolabs PURExpress^®^ *In Vitro* Protein Synthesis Kit) was purchased from Byasis (Mexico) [35S]-Methionine was purchased from PerkinElmer (Waltham, MA, United States). All other reagents were from Sigma-Aldrich (St. Louis, MO, United States).

### DNA Manipulations

All Ankyrin repeat Domain of the *Drosophila* Notch Receptor (PDB code: 1ot8) constructs were cloned in a previously described pET19b plasmid (Nilsson et al., 2015) (Novagen, Madison, WI, United States). The full-length in vitro-translated construct, and truncations thereof, with different linkers under the control of a T7 promoter, were composed by the following elements (see Figure 3 panel B, and Supplementary Table S1): 1) an unstructured N-terminal segment (154 residues) from *Escherichia coli* LepB (to facilitate visualization by SDS-gel electrophoresis for short constructs); 2) GSGS … SGSG-flanked the Ankyrin repeat; 3) a short unstructured linker derived from LepB composed by different lengths; 4) the 17 residues *Escherichia coli* SecM AP arrest peptide (sequence FSTPVWISQHAPIRGSP) and 5) a 23-residue long LepB-derived C-terminal tail (to ensure that the arrested and full-length forms of the protein can be separated by SDS-PAGE).

The full-length Ankyrin repeat construct was used as a starting point to create a library of 47 constructs. In a first step, we removed residues from the linker (L). In a second step we remove residues from the Ankyrin repeat protein by keeping constant linker of 8 residues + *E. coli* SecM AP arrest peptide (17 residues). We generated full length band controls (to define the identity of the bands in SDS-gels) for selected (L) by mutating the last proline of the arrested peptide to Alanine. Also, we generated arrested band controls by mutating the last proline of the arrest peptide to stop codon. The mutagenesis primers were designed with the program *AAscan (* *Sun et al., 2013* *)*. The mutagenesis procedure was done by an adaptation (Zheng et al., 2004) of the original QuickChange™ (Agilent Technologies, Santa Clara, CA, United States) site-directed mutagenesis protocol.

We generated linear DNA PCR products for *in vitro* expression, these were treated with DpnI (New England Biolabs). Chemically competent Dh5alpha *E. coli* cells were transformed and plated onto LB agar plates supplemented with ampicillin. Single colonies were picked to inoculate overnight cultures from which plasmids were subsequently purified. All constructs were verified by sequencing (MCLAB, 320 Harbor Way, South San Francisco, CA 94080, United States).

### *In vitro* Transcription and Translation

Linear DNA constructs were generated from purified plasmids by PCR using primers overlapping the T7 promoter and terminator and were purified prior to *in vitro* transcription and translation (PCR Purification Kit, Qiagen).

*In vitro* transcription and translation was performed using the NEB PURExpress *In Vitro* Protein Synthesis Kit, with the purified PCR products as templates. Synthesis of [35S]-Met labeled proteins, was performed in an Biosan thermomixer at 37°C, 500 rpm, for 15 min. The reaction was stopped by the addition of 1: 1 volume of 20% ice-cold TCA. The samples were incubated on ice for 30 min and centrifuged for 5 min at 21,000 g at 4°C. Supernatant was discarded and pellets were dissolved in sample buffer and treated with RNase A (400 μg·mL−1) for 15 min at 37°C before being resolved by SDS/PAGE.

### Quantitation

Proteins were separated by SDS/PAGE and visualized on a Typhoon GE FLA-9500 phosphoimager. The bands were quantified to estimate the fraction full-length protein fFL = IFL/(IFL + IA), where IFL is the intensity of the band corresponding to the full-length protein, and IA is the intensity of the band corresponding to the arrested form of the protein (detailed procedure provided in Supplementary Figure S5). Bands were quantitated using ImageJ (http://rsb.info.nih.gov/ij/) to obtain an intensity cross section, which was subsequently fitted to a Gaussian distribution using an in-house software. All experiments were done at least in duplicates (standard deviations from independent measurements are shown, Figure 3 panel A).

## Results

### Sequence Based Comparison of the Notch Repetitive Protein

We used as query a hidden Markov model (HMM) representing the sequence of the Ankyrin Notch (Zweifel et al., 2003) to search the SCOP95 (the SCOP domains filtered at 95 percent redundance) database of HMM profiles. According to HHsearch documentation hits with >30% probability are worth considering as homologous hits (at least locally). HHsearch can detect homologous relationships below 20% sequence identity (the twilight zone of sequence comparisons). Therefore, sequence identity and/or E-value could not be an appropriate measure of relatedness anymore.

**FIGURE 2:**
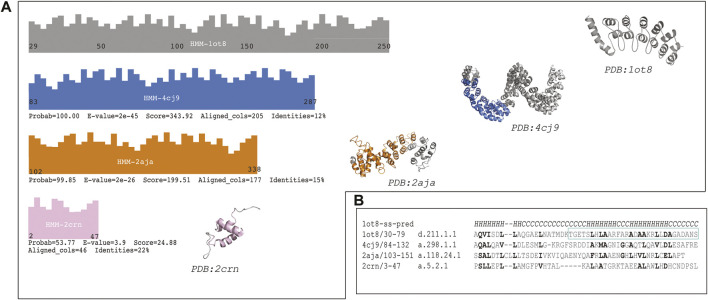
The sequence of the Ankyrin Domain of the *Drosophila* Notch Receptor was used to build a Hidden Markov Model (HMM) to search the SCOP95 database of proteins also represented by HMMs. **(A)** Graphical representation of the hits from different folds (according to SCOP); three high probability hits are shown (probability better than 50%). Fold in blue: **(A)**298: Left-handed alpha-alpha superhelix, which is a TAL (transcription activator-like) effector. The hit covered 205 columns (left) and the region aligned in the protein is shown in blue on the structure (right). Fold in orange: **(A)**118: alpha-alpha superhelix which is a Hypothetical protein LPG2416 RuVZ. The hit covered 177 columns (left) and the region aligned in the protein is shown in orange on the structure (right). Fold in pink: **(A)**5: RuvA C-terminal domain-like (3 helices; bundle, right-handed twist), which is a UBA domain. The hit covered 46 columns (left) and the region aligned in the protein is shown in pink on the structure (right). **(B)** Multiple sequence alignment of a common region hit in the query (1ot8) by all the three previously mentioned folds. Similar or identical residues are shown in bold, and the second repeat of the Ankyrin domain is shown with a cyan rectangle (Supplementary Figure S2).

The first hit (probability = 100.00, E-value = 2e-45 and 205 aligned columns) was the TAL transcription activator-like effector from *Burkholderia rhizoxinica*: (Fold: a.298, Left-handed alpha-alpha superhelix), see Figure 2 panel A). This protein displays a DNA binding function which is similar to the protein binding function of the Ankyrin repeat. The alignment covers more than 10 helices in both proteins (see Supplementary File S2, raw HHsearch output). This protein structure is also a repetitive solenoid with left-handed bundles that associate in a right-handed superstructure composed by 33 aa repeats. The repeats in query and match (1ot8 and 4cj9) proteins aligned well (Figure 2 panel b). The loops in the repeats in this match (4cj9) determine the specificity for the DNA interaction, similarly to the loops present in the ankyrin repeat (1ot8) domain mediate protein binding.

The second hit (probability = 99.85, E-value = 2e-26 and 177 aligned columns) from a different fold is a binding protein from *Legionella pneumophila*: (fold a.118, alpha-alpha superhelix). The alignment covers almost 10 helices with several secondary structure matches. This fold is likely to be homologous to the ankyrin repeat given that parts of the fold are similar in assembly and sequence to the Ankyrin repeat protein. However, the topology is not identical in different areas of the protein, and the sequence identity is low. This protein also displays a binding function.

The last hit from a different fold was (probability = 53.77; E-value = 3.9 and aligned columns = 46) the Suppressor of T-cell receptor signaling (Figure 2 panel A) from *Homo sapiens* (Fold: RuvA C-terminal domain-like). The protein is a 3-helical bundle with a right-handed twist. This domain facilitates polyubiquitin chain formation, seems to interact in a regioselective manner with ubiquitin. The protein-protein interaction function could be analogous to the ankyrin repeat binding function as well. In this case three helices are aligned between the query and the match.

The distribution of the hits is depicted in Figure 2. Where the two first hits are covering a long region of the Ankyrin domain the last one is confined to the N-terminal part of the query. A detailed multiple sequence alignment showing a common region of all proteins is showed in Figure 2, panel B (Supplementary Figure S2). Similar and/or identical residues in sequence are highlighted. The similarities among the proteins are more marked as the probability is higher, and they fade out as the probability gets lower.

These high-probability hits among very divergent proteins (classified as different folds) might indicate the presence of a folding unit conserved through evolutionary time (Supplementary Figure S2) that was used by nature to tinker thereby creating different architectures. To explore how the sequential removal of the shared theme would affect the stability of the full ankyrin repeat protein architecture we decided to experimentally characterize its folding on the ribosome. The vectorial nature of protein folding in the ribosomal exit tunnel, where the N-terminal side of the protein could start to fold while the rest is still being translated, posed an excellent experimental set up to look for periodic folding events of the repetitive units of the Ankyrin Notch domain.

### Force Profile Analysis of Cotranslational Folding

Currently we know that several mechanical forces are at play during translation (Leininger et al., 2019a). These forces can be accurately measured on the ribosome by using force sensors based on translational arrest peptides (APs) (Nilsson et al., 2015). APs are short polypeptide fragments that block, or slow down translation while being synthetized on the ribosome. The sensors were initially developed by the von Heijne laboratory (Cymer et al., 2015) but nowadays have been applied by other researchers (Marsden et al., 2018; Liutkute et al., 2020). The SecM translational arrest peptide (AP) interacts with the exit tunnel to stall translation when the ribosome sits at the last proline of the AP. The arrest can be released by external pulling forces on the nascent chain produced by a range of cotranslational processes such as protein folding (Kemp et al., 2020).

**FIGURE 3:**
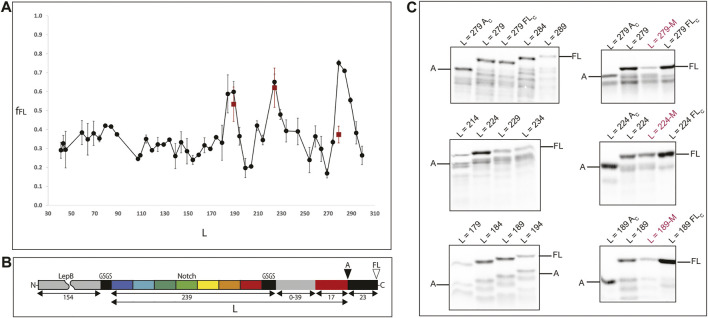
Experimental determination of protein folding on the ribosome. **(A)** Force profile generated by the full protein and a set of C-terminal truncations of the Ankyrin Notch. Shorter constructs have C-terminal deletions in the Ankyrin domain (keeping a constant linker) and longer constructs have linker segments of 0–39 residues (gray rectangle in panel B). All points are at least two independent measurements; averages ±SE are shown. A double mutation (A125E/A126F) known to disrupt folding for the full protein was introduced at the peak constructs (red squares). **(B)** The force-generating ankyrin domain (and C-terminal truncations thereof; repeats indicated in colors) is connected, via a variable-length linker (gray) and an “insulating” SGSG tetrapeptide (black), to the 17-residue SecM AP (red). An N-terminal 154-residue segment from the E. *coli* LepB protein (gray) and a short GSGS segment (black) is included in short constructs (length L ≤ 110, where L is the number of residues between the N-terminal end of the Ankyrin part and the last residue in the AP) in order to make short Ankyrin constructs conveniently amenable to analysis by SDS-PAGE, and a 23-residue C-terminal tail (also from LepB) is appended at the C terminus in order to make it possible to separate arrested **(A)** and full-length (FL) chains by SDS-PAGE. The sequences of all constructs are included in Supplementary Table S1. **(C)** SDS-PAGE images of measurements around peak constructs and folding disrupting mutations. Left: gels showing expression of constructs around the *f*_*FL*_ peaks [L = 279, L = 224, L189]. The top panel left shows two controls around the L = 279 measurement: L = 279 Ac (same construct with a stop codon at position P17 in the arrest peptide) and L = 279 Fc (inactivated arrest peptide P17A mutation) to identify the bands to be integrated during the measurements. Right: measurements of mutations M (A125E/A126F) at the peak constructs (indicated in red) also flanked by controls.

To study the folding on the ribosome of the full ankyrin repeat protein (and C-terminal truncations thereof) we designed a translational fusion (Figure 3, panel B) with the following elements: 1) unstructured segment of the LepB protein (154 aa) for visualization of translational fusions <10 kDa in SDS-PAGE (L < 110), 2) the ankyrin repeat protein (239 aa) flanked by two insulating GSGS linkers, 3) a segment of the LepB protein used as rigid linker L (39 aa), 4) the arrest peptide SecM from *E. coli* (17aa), and 5) a short C-terminal extension (23aa).

Next, we produce the force profile of the full Ankyrin protein (Figure 2, panel A) by removing residues (in 5 aa steps) from the LepB linker (initially composed by 39 aa) up to a linker of L25 (8 aa from the LepB linker plus 17 aa from the SecM arrest peptide). In a second stage, to measure truncated versions of the protein we removed residues from the C-terminal end of the ankyrin repeat protein in 5 residues steps. We produced and sequenced 47 constructs that were expressed for 15 min at 37°C with the PURE express *in vitro* translation system (Shimizu et al., 2001). We visualized the ^35^S-Met radiolabeled products in SDS-PAGE and measured the intensities of the bands for the expressed products. For every construct, we recorded the presence of two bands separated by a 23 aa difference in size. First, the full-length band (FL): were the nascent chain folds and the chemical energy transduced into mechanical force to the ribosomal PTC (ΔG°- > pN) thereby alleviating the arrest and allowing the ribosome to translate the full-length construct (see Supplementary Figure S3).

Second, an arrested band (A): where the nascent chain did not fold and therefore did not produce enough force to release the arrest; this in turn does not allow the expression of the 23 aa terminal extension but only up to the last proline of the arrest peptide SecM (Figure 2, panel C). We calculated the fraction full-length of the protein, *f*_*FL*_ = I_FL_/(I_FL_ + I_A_), where I_FL_ is the intensity of the full length band, and I_A_ is the intensity of the arrested band. *f*_*FL*_ is a measure of the force exerted on the AP during translation.

### The Ankyrin Force Profile in Context of Classical *in vitro* Folding Studies

The *in vitro* folding kinetic and thermodynamic properties of the ankyrin repeat protein have been meticulously characterized by the Barrick laboratory. With an elegant set of experiments they experimentally determined the folding landscape of the ankyrin repeat protein (Mello and Barrick, 2004). The overall thermodynamic stability of the full seven repeat protein (named Nank1-7) was in the range of ΔG° = -6.65 kcal·mol-1.

They determined stabilities for shorter versions of the Notch Ankyrin protein by removing one (creating Nank1-6, ΔG° = -2.85 kcal·mol-1) and two repeats (creating Nank1-5, ΔG° = -2.69 kcal·mol-1) from the C-terminal end of the protein. Moreover, they used the stabilizing osmolyte trimethylamine N-oxide to obtain true thermodynamic parameters for a version containing only four repeats (named Nank1-4); remarkably, this truncated version folded with ΔG° = +0.37 kcal·mol-1. In another publication (Zweifel et al., 2003) they created a double mutant (A125E and A126F) of the seven repeat protein (Nank1-7) that was completely destabilized (ΔG° ≅ 0) (Supplementary Table S2).

Previously (Farias-Rico et al., 2018) we found a linear correlation between the thermodynamic stability (ΔG°) measured *in vitro* for a set of a ribosomal protein S6 mutants (Haglund et al., 2008) and the *f*_*FL*_ measured with Force Profile analysis (FPA) on the ribosome. Alternatively, Leininger et al. (Leininger et al., 2019b) with computational work demonstrated that force on the nascent chain increases with increasing thermodynamic stability in a sigmoidal manner. In a recent perspective article (Leininger et al., 2019a) by using a sigmoidal fit and subtracting the destabilizing effect of the ribosome (Samelson et al., 2016) to the data that we have previously produced (Farias-Rico et al., 2018), Leininger and coauthors conciliated experimental observations and concluded that the fraction of full-length protein (*f*_*FL*_) is a function of the probability that the domain is folded at a fixed distance from the ribosome (L).

In this work we observed a main *f*_*FL*_ = 0.75 peak at L = 279; this construct corresponds to the full ankyrin repeat protein tethered to the ribosome with a LepB linker (23 aa) and the arrest peptide SecM (17 aa). Thus, the C-terminal end of the protein is located at 40 aa away from the peptidyl transferase center (PTC) of the ribosome. Next, we observed a second *f*_*FL*_ = 0.65 peak at L = 224 and a minor *f*_*FL*_ = 0.59 peak at L = 189. These two peaks correspond to the deletion from the ankyrin protein of 1½ and 2½ repeats respectively. The C-terminal ends of both truncations are tethered to the ribosome by a short linker (8 aa) plus the SecM arrest peptide (17 aa). And finally, we observed a slight continuous increase in the overall *f*_*FL*_ between L30 and L90, this region of the force profile would correspond to very short versions of the ankyrin protein, equal to one or less repeats.

In full agreement with the *in vitro* results obtained by the Barrick laboratory we find a linear correlation between the *f*_*FL*_ of the three peaks (L = 279; L = 224; L = 189) and the ΔG° of the Nank1-7, Nank1-5, and Nank1-4 constructs (Supplementary Figure S4). It is not clear if a sigmoidal fit, previously suggested by Leininger and coauthors, would better describe the data because we only have three points. Also, we cannot account for the destabilizing effect of the ribosome, since the ΔG° for the Nank1-4 is already positive. The Barrick lab increased the overall thermodynamic stability of the Ankyrin repeat protein by adding consensus repeats (Tripp and Barrick, 2007); it would be interesting to measure the folding of these versions on the ribosome to test Leininger et al. assumptions of a sigmoidal fit plateau in the mechanical force exerted on the ribosome by the nascent chain.

In order to test whether the peaks correspond to folded Ankyrin versions we introduced the double mutation M2 (A125E and A126F) (Zweifel et al., 2003) to the three different peak constructs (L = 279; L = 224; L = 189). The *f*_*FL*_ measurements associated with these mutated constructs are shown as red squares in the force profile at L279, 224 and 189 (Figure 2 panel A, C and Supplementary Table S2). We observed a dramatic reduction in the *f*_*FL*_ of the full-length construct (L279, from 0.74 to 0.37) and no major difference for the other peaks.

## Discussion

In this work we used as query an Ankyrin repeat protein to perform a deep sequence-based search with state-of-the-art tools for homology detection (Hidden Markov Models, or HMMs). We found high probability hits with three proteins classified as different folds. The aligned region common to all hits covers the repetitive 33-residue motif consisting of two alpha helices separated by loops that form a hairpin like β-sheets with the neighboring loops in the full ankyrin structure. Our analysis shed light on how this 33-residue motif, present 7 times in the full ankyrin repeat protein, is also used as building block by nature to generate other protein folds over long evolutionary timescales.

In general, there is not a consensus explanation for the conservation of these fragments among proteins from different folds (Farias-Rico et al., 2014; Alva et al., 2015). It is common knowledge that sequence conservation among proteins from the same family is due to functional reasons. In the case of different folds it has been argued that conservation could be related with rudimentary binding (Ferruz et al., 2021). Also, rudimentary helicase like activities have been shown for 40 residues long peptides (Vyas et al., 2021). However, there are many instances of conserved short sequences that have no apparent function. Looking for alternative explanations, we observed that the ribosomal tunnel can accommodate up to 35–40 residues in an extended conformation; interestingly, this is the size of most shared ancestral fragments (Alva et al., 2015). Perhaps the well-documented confinement effect provided by ribosomal exit tunnel is playing a role in the evolutionary selection of fragments-foldons.

**FIGURE 4:**
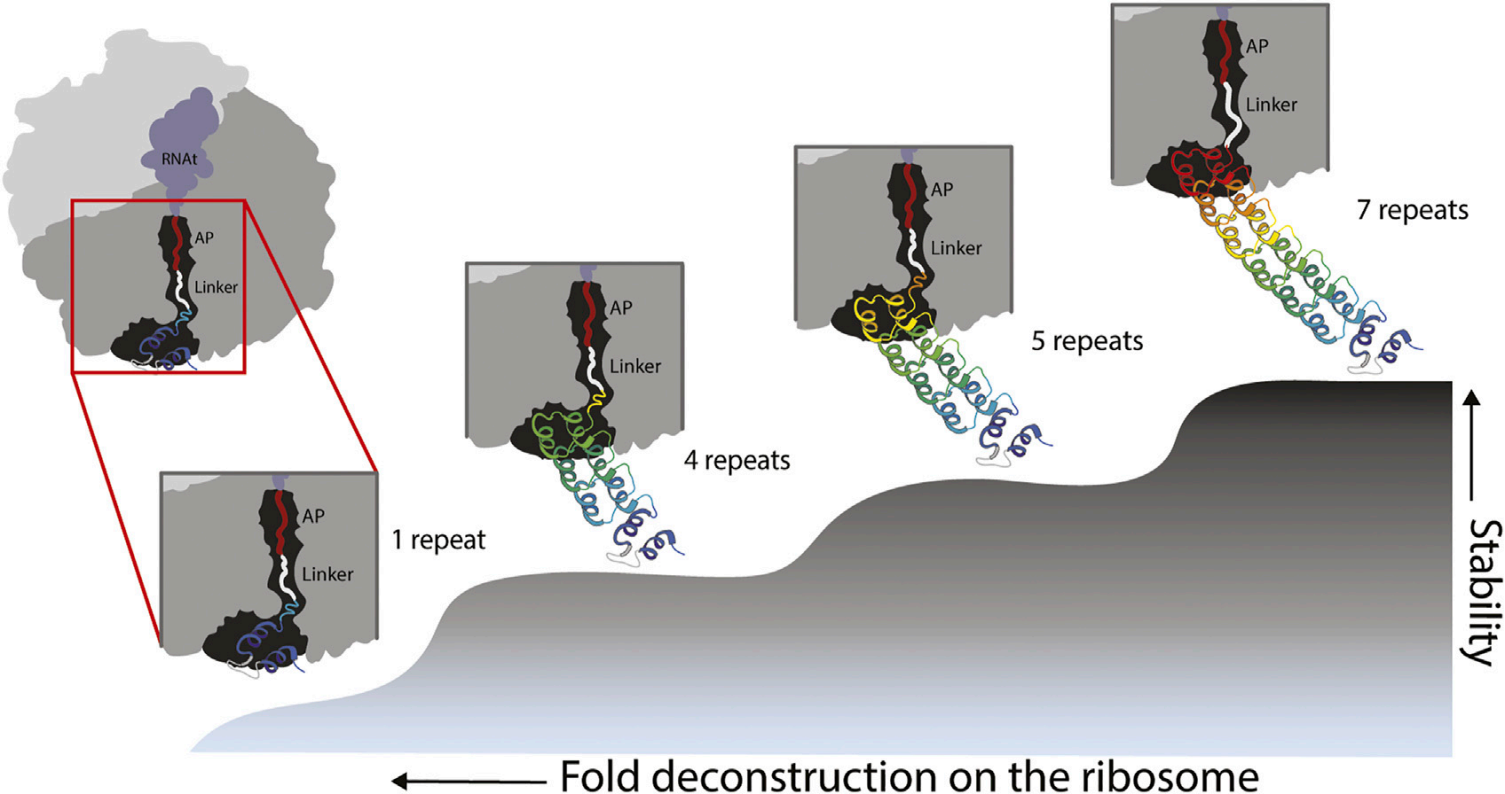
Model of fold deconstruction on the ribosome. The Ankyrin Domain of the *Drosophila* Notch Receptor might have arisen by duplication and fusion of a single repeat. Four probable compacted/folded states are shown, the last three states correspond to peaks in the force profile: L-279 = 7 repeats, L-224 = 5 repeats and L189 = 4 repeats. According to the force profile of Figure 3, the full-length protein folds at L40 from the peptidyl transferase center of the ribosome (L-279). It is the same case for truncated versions with 4 and 5 repeats. During the truncation process of the version with 5 repeats for instance, 1/2 sixth repeat (orange) becomes an unstructured linker because it cannot intrinsically fold due to missing resides within the repeat. It is the same case for the 4 repeats version of the protein. In the case of an ancestral state represented by a single repeat, the force profile shows a modest, and somehow flat constant increase in fFL, this might reflects pulling force exerted by an unstructured single repeat. It is unlikely a single repeat would produce a defined peak due its intrinsic instability. However, in early stages of protein evolution, a proto-exit tunnel might have promoted the formation of super secondary structures such as the repeats of the Ankyrin domain.

Our experiments showed folding of the full ankyrin repeat protein during translation when its C-terminal end is located at ∼ 40 residues (L = 23 aa linker +17 aa SecM AP) away from the PTC. By removing 1½ and 2½ repeats from the C-terminal end of the full protein we created versions with 5 and 4 full repeats. Both versions were also carrying a ∼16 residues tail from the truncated ½ repeat. These versions were tethered to the ribosome by a linker of L = 25 (8 aa linker +17 aa SecM-AP). We hypothesized that the ½ repeat left in these truncated versions became unstructured and played the role of an extended linker adding up to ∼40 residues. If this is the case, the truncated versions also fold when their C-terminal end is located at ∼ 40 residues from the PTC (Figure 4). These findings are in line with several works (Goldman et al., 2015; Samelson et al., 2016; Farias-Rico et al., 2018; Cassaignau et al., 2020) that extend the influence of the ribosome on the folding of proteins up to ∼40–50 residues away from the PTC, while smaller proteins can fold right at the vestibule or inside the tunnel (Farias-Rico et al., 2018; Tian et al., 2018; Wruck et al., 2021).

The interaction between the ribosome and the full-length repeat protein must be different than the interactions with the truncated versions because the incomplete versions display exposed inter-repeat surfaces. We recorded the folding of a 5-repeat protein at L = 224, this truncation produced less force than the full-length protein (corresponding to a lower ΔG°). The removal of 2½ repeats poses a similar scenario at L = 189. At this *L* we observed the compaction of the 4-repeat truncation with *f*_FL_ = 0.59.

Surprisingly in this case, *in vitro* studies with the purified 4-repeat protein have shown +ΔG° under a specific set of experimental conditions (Mello and Barrick, 2004). The authors also mentioned that interactions between the repeats are more important than the stability of each repeat. In agreement with such observation, we did not see a clear peak in pulling force for the constructs that correspond to a single repeat (L = 74) but only for constructs constituted by at least 4 repeats. For the 4 and 5 repeat constructs, the ribosome could be interacting with the exposed surfaces via electrostatic interactions (Wruck et al., 2021) or unspecific binding (Cassaignau et al., 2021). One could argue on the contrary, that the full protein is a completely folded domain with no available surfaces to establish productive interactions with the ribosome; therefore, its stability could be negatively affected. We propose therefore, that the effect of the ribosome on thermodynamic stability of the nascent chain could be determined by its relative position and the composition of its surface exposed lateral chains.

To confirm a folding event for the three peaks observed in the full force profile we engineered the double mutation M2 (A125E and A126F). The introduced M2 mutation, which is located at the center of the fourth repeat, resulted in a major reduction of *f*_*FL*_ for the major peak at L279 because the differences in free energy between the folded and the unfolded state were major. But for the smaller peaks at L189 and L224 the influence of the mutation might be negligible because these versions are already unstable of fold with low energy. In this case the ribosome could be mimicking the role of the osmolyte trimethylamine N-oxide in stabilizing the 4-repeat construct. It has been established that evolution tunes proteins to be just marginally stable under cellular conditions, and probably most proteins in the cell could be metastable (Sorokina and Mushegian, 2018). For instance, the unfolded conformation of the alpha-lytic protease is more stable than its native counterpart (Sohl et al., 1998). So it is not surprising that binding type interactions between the ribosome and the Nank1-4 construct could stabilize this version of the full Ankyrin repeat protein.

If the ribosomal exit tunnel in fact promotes the compaction of foldons, which in solution would not fold due to the differences in conformational entropy of the two microscopic environments, one could expect to detect a clear folding peak for a single Ankyrin repeat deep in the ribosomal tunnel. We did not see any clear evidence of a folding peak. We saw however, a slight continuous increase in *f*_*FL*._ This could be explained by experiments (Leininger et al., 2019a) that demonstrate pulling forces exerted by unstructured chains. Another explanation could be that our current experimental set up did not allow us to clearly demonstrate the folding of the single theme on the ribosome. But the role of this theme in the evolution of the fold was clearly demonstrated by deconstructing the ankyrin repeat protein and showing folding peaks only when full repeats/themes are removed (Figure 4).

With site directed mutagenesis in every repeat, the Barrick laboratory demonstrated no folding for the first repeat of the Notch. A conserved 15 aa insertion was proposed as the source of its low sensibility to mutations that clearly disrupt the folding of the other six repeats (Bradley and Barrick, 2006).

Yet, even by removing the insertion, it is unlikely that a single repeat would fold on the ribosome, because other studies from the Barrick laboratory calculate a ΔG° = +5.5 kcalmol energy of folding for a single repeat. It would be interesting to engineer a biding site for a metal in the first repeat to see if we record robust pulling force.

We demonstrated how novel homology algorithms for homology detection could be used to look for folding intermediates. It can be conceptually complicated to imagine how evolution could have produced such intermediates on the ribosome. For example, it has been proposed that at the origin of the folded proteins the ribosomal exit tunnel played an important role by allowing the sampling of conformations (Kovacs et al., 2017) that later became folded or became a part of a bigger folded architecture. Early peptides originally folded around RNA and as the tunnel grows longer, more complex architectures could be sampled. Alternatively, RNA binding could allow smaller conformational entropy within the exit tunnel. Finally, we envision a future where the exit tunnel is conceptualized as a novel active site under evolutionary pressure to select for the suitable choreography of translation and folding.

## Data Availability

The original contributions presented in the study are included in the article/Supplementary Material, further inquiries can be directed to the corresponding author.
