# Supplementary material for: Folding and Evolution of a Repeat Protein on the Ribosome
Source: Front Mol Biosci. 2022 May 30;9:851038. doi: 10.3389/fmolb.2022.851038 (PMC9189291; doi:10.3389/fmolb.2022.851038)
Supplement: Supplementary file 1 [file DataSheet1.PDF]

## Supplementary figure 1. Raw HHsearch output (fragment) for the hits discussed in main text.

## Full *HHsearch* output is available as supplementary file 2.

```
Query         d1ot8a_ d.211.1.1 (A:) Neurogenic locus notch receptor domain {Fruit fly (Drosophila melanogaster) [TaxId:
7227]}
Match_columns 209
No_of_seqs    234 out of 19272
Neff          7.8
Searched_HMMs 30201
Date          Wed May  2 13:03:39 2018
Command       /scratch/s2angags/hhsuite-2.0.16-linux-x86_64/bin/hhsearch -Z 10000 -B 10000 -cpu 8 -i
/scratch/s2angags//scope95_2.07_psi_hhm/d1ot8a_.hhm -d /scratch/s2angags/scope95_2.07_hhmdb -o
/scratch/s2angags//scope95_2.07_psi_hhs/d1ot8a_.hhs -v 2 -norealign -ssm 0

No 69
>d4cj9a_ a.298.1.1 (A:) BurrH {Burkholderia rhizoxinica [TaxId: 412963]}
Probab=100.00  E-value=2e-45  Score=343.92  Aligned_cols=205  Identities=12%  Similarity=0.060  Sum_probs=0.0

Q ss_pred              HHHHHHHHHCCCCCCccCCCCCCCHHHHHHHcCCHHHHHHHHHcCCccCcCCCCCCCchhHhhHHhhhhhhhhhHhhccc
Q d1ot8a_         2 AQVISDLLAQGAELNATMDKTGETSLHLAARFARADAAKRLLDAGADANSQDNTGRTPLHAAVAADAMGVFQILLRNRAT   81 (209)
Q Consensus       2 ~eiv~~Ll~~Ga~vn~~~d~~G~T~Lh~Aa~~g~~~~v~~Ll~~Gadvn~~d~~g~TpLh~A~~~~~~~~iv~~Ll~~~~~   81 (209)
                    .++|+.||++|+++|  ..+.+|.||||+||++|+.++|++|+++|++++.+|..|.||||+|+.+|+.++|+.|++.++
T Consensus     83 ~~~v~~Ll~~g~~~~~-~~~~~g~tpL~~Aa~~g~~~~v~~Ll~~~a~~~~~~~~~~~~~t~L~~A~~~g~~~~v~~Ll~~ga-  160 (752)
T d4cj9a_       83 AQALQAVLDLESMLG-KRGFSRDDIAKMAGNIGGAQTLQAVLDLESAFRERGFSQADIVKIAGNNGGAQALYSVLDVEP-  160 (752)
T ss_pred              HHHHHHHHHcCcccc-cCCCCHHhHHHHHhhcCcHHHHHHHHHcCCCcccccCCCHHHHHHHHccCCHHHHHHHHHhhcc-


Q ss_pred              ccccccccCChhHHHHHHHHHHhccccccccCcCcccccccccchhHHHHHHHhcchhHHHHHHhHHhccccccccccCCCchh
Q d1ot8a_        82 NLNARMHDGTTPLILAARLAIEGMVEDLITADADINAADNSGKTALHWAAAVNNTEAVNILLMHHANRDAQDDKDETPLF  161 (209)
Q Consensus      82 d~~~~~~~~g~TpL~~A~~~g~~~~v~~Ll~~gadvn~~d~~g~T~Lh~Aa~~g~~~iv~~Ll~~ga~~n~~d~~g~TpLh  161 (209)
                    +++.++..|.||||+|+..|+.+++++|+++|++++..+..+.||||+|+.+|+.+++++|+++|+++|.++..+.||||
T Consensus    161 ~~~~~~~~~~~~~t~l~~A~~~~g~~~~~v~~Ll~~~a~~~~~~~~~~~~~~~~~l~~Aa~~g~~~~~v~~Ll~~g~~~~~~~~~~~~~~~t~L~  240 (752)
T d4cj9a_      161 TLGKRGFSRADIVKIAGNTGGAQALHTVLDLEPALGKRGFSRIDIVKIAANNGGAQALHAVLDLGPTLRECGFSQATIAK  240 (752)
T ss_pred              ccccCCCCccchHHHHhccCccceeeeeccccccchhcCCCCHHHHHHHHHhcCChhheeEeeccCccccCCCCCcccHhH


Q ss_pred              hhccccHHHHHHHHHCCCCCCCCCCCCCCHHHHHHHcCCHHHHHHhh
Q d1ot8a_       162 LAAREGSYEASKALLDNFANREITDHMDRLPRDVASERLHHDIVRLL  208 (209)
Q Consensus     162 ~A~~~g~~~~~v~~Ll~~ga~~~~~~~~d~~g~tpL~~A~~~~g~~~~vv~~L  208 (209)
                    +|++.|+.++|++|+++|++++.++..+.||||+|+..|+.++|++|
T Consensus    241 ~Aa~~g~~~~vv~~Ll~~ga~~n~~~~~~~~~~t~L~~Aa~~g~~~~v~~L  287 (752)
T d4cj9a_      241 IAGNIGGAQALQMVLDLGPALGKRGFSQATIAKIAGNIGGAQALQTV  287 (752)
T ss_pred              HHhhcchhhhhhhhHhhcCCCcccccCccHHHHHHHHHhccCccchHHHHH

No 104
>d2ajaa1 a.118.24.1 (A:3-348) Hypothetical protein LPG2416 {Legionella pneumophila [TaxId: 446]}
Probab=99.85  E-value=2e-26  Score=199.51  Aligned_cols=177  Identities=15%  Similarity=0.097  Sum_probs=0.0

Q ss_pred              CHHHHHHH--HHCCCCCCccCCCCCCCHHHHHHHcCCHHHHHHHHHcCCCc--cCcCCCCCCCchhHhhHHhhhhhhhhhH
Q d1ot8a_         1 TAQVISDL--LAQGAELNATMDKTGETSLHLAARFARADAAKRLLDAGAD--ANSQDNTGRTPLHAAVAADAMGVFQILL   76 (209)
Q Consensus       1 s~eiv~~L--l~~Ga~vn~~~d~~G~T~Lh~Aa~~g~~~~v~~Ll~~Gad--vn~~d~~g~TpLh~A~~~~~~~~iv~~Ll   76 (209)
                    ..+++++|  +....++-...+.+|+||||+||++|+.++|++|+++|++  .+..+.+|+||||+|+++||.+||++|+
T Consensus    102 ~~~~~~~~~L~~~~~~~~~~~~~~~~~~~~~~~a~~~Aa~~Ghl~vvk~L~~~~~~~~~~~~~~~~~~~~~~~~~t~l~~Aa~~Gh~~iv~~Ll  181 (346)
T d2ajaa1      102 CSSALDTLCLLLTSDEIVKVIQAENYQAFRLAAENGHLHVLNRLCELAPTEIMAMIQAENYHAFRLAAENGHLHVLNRLC  181 (346)
T ss_pred              ChHHHHHHHhcChHHHHHHHHccchHHHHHHHHcCCHHHHHHHHHHCCHHHHHHHHHcCCCcHHHHHHHHhcCCHHHHHHHHH


Q ss_pred              hhcccccccc-cccCChhHHHHH-HHHHhcccccccCcCccccccccchhHHHHHH----------------------
Q d1ot8a_        77 RNRATNLNAR-MHDGTTPLILAA-RLAIEGMVEDLITADADINAADNSGKTALHWAA----------------------  131 (209)
Q Consensus      77 ~~~~~d~~~~-~~~g~TpL~~A~-~~g~~~~v~~Ll~~gadvn~~d~~g~T~Lh~Aa----------------------  131 (209)
                    +.++..+... +.+|.||+++|+ ++|+.++++++|++.|++++..+..+.+..|..+
T Consensus    182 ~~~~~~~~~~~~~~~~~~~~~~~~~~~~al~~Aa~~~gh~~iv~~Ll~~~~~~~~~~~~~~a~~~~~~~~~g~~~v~~~~~~~~~~~~~~~~~~l~~~~~~~~~~~~~~~~~~~  261 (346)
T d2ajaa1      182 ELAPTEATAMIQAENYYAFRWAAVGRGHHNVINFLLDCPVMLAYAEIHEFEYGEKYVNPFIARHVNRLKEMHDAFKLSNP  261 (346)
T ss_pred              HhCCCcHHHhhHhhcCCCchhhHHHHHhHHHHcCCccchhHHHHHHHHHhHCchhHHHHHHHHHHHHHHHHHHHHHHHHhcCC


Q ss_pred              ---------------------------HhcchhHHHHH-----hHHhccccccccccCCCchhhhcccccHHHHHHHHH
Q d1ot8a_       132 --------------------------AVNNTEAVNIL-----LMHHANRDAQDDKDETPLFLAAREGSYEASKALLD  177 (209)
Q Consensus     132 --------------------------~~g~~~iv~~L-----l~~ga~~n~~d~~g~TpLh~A~~~g~~~~~v~~Ll~  177 (209)
                                              ..+..+.+++|     ++..++.+..+..|.||||+||+.|+.++|++||+
T Consensus    262 ~~~~~~~~~~~~~~~~~~~~~~~~~~~~~~~~~li~~~~~~~~~~~~~~~~l~~ll~~i~~vk~l~~~~~~~~~~~~~~~~~~nt~LhlA~~~Gn~~iv~lLLs  338 (346)
T d2ajaa1      262 DGVFDLVTKSECLQGFYMLRNLIRRNDEVLLDDIRFLLSIPGIKALAPTATIPGDANELLRLALRLGNQGACALLLS  338 (346)
T ss_pred              CceeeccccccHHHHHHHHHHHHHHHHcCChhHHHHHHHHHhChHHHHHHhcCCCCCCCCCCcCHHHHHHHHhCCHHHHHHHhc

No 105
>d2crna1 a.5.2.1 (A:8-58) Suppressor of T-cell receptor signaling 2 (STS-2) {Human (Homo sapiens) [TaxId: 9606]}
Probab=53.77  E-value=3.9  Score=24.88  Aligned_cols=46  Identities=22%  Similarity=0.302  Sum_probs=0.0

Q ss_pred              CHHHHHHHHHCCCCCCccCCCCCCCHHHHHHHcCCHHHHHHHHHcCCccCc
Q d1ot8a_         1 TAQVISDLLAQGAELNATMDKTGETSLHLAARFARADAAKRLLDAGADANS   51 (209)
Q Consensus       1 s~eiv~~Ll~~Ga~vn~~~d~~G~T~Lh~Aa~~g~~~~v~~Ll~~Gadvn~   51 (209)
                    |+++|+.|++.|...+...     -+|...--.+-..++++|+++.-|++.
T Consensus      2 np~~l~~L~~MGF~~~~a~~-----~~AL~~t~n~~ve~A~~~Wl~~h~~d~~~   47 (51)
T d2crna1        2 SPSLLEPLLAMGFPVHTAL-----KALAATGRKTAEEALAWLHDHCNDPSL   47 (51)
T ss_pred              CHHHHHHHHHCCCCHHHHH-----HHHHhCCCCCHHHHHHHHHHcCCCCCC
```

**Supplementary figure 2. PDB coordinates for the second repeat aligned in 1ot8 to other proteins detected by the HHsearch comparisons (main figure 2, cyan box).** Ribbon representation (red = helix, green = coil). The HHsearch profile-profile alignments are longer for every hit, and they are inclusive of the aligned repeat (highlighted in color in the raw HHsearch alignments).

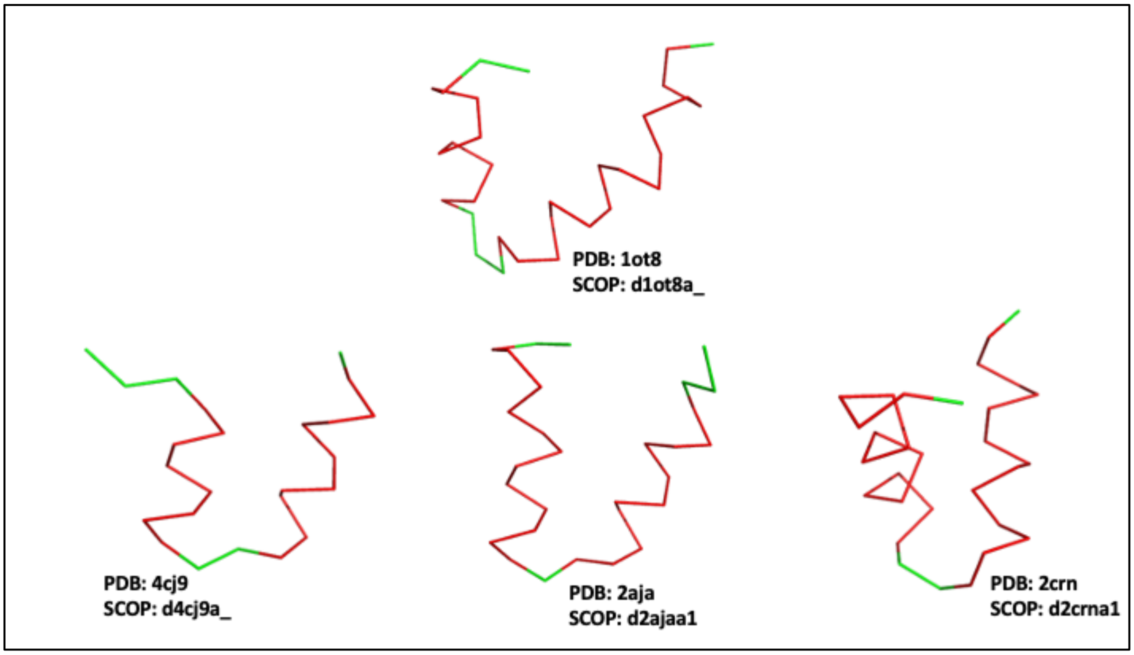


**Supplementary Table 1**

Aminoacid sequences of [Lep154]-GSGS-1OT8-SGSG-Linker-Arrest-peptide-Extension-c-terminal constructs analyzed in the work. Note that the N-terminal [Lep159]-GSGS is only present in constructs with L<110 (<10 kDa) to better resolve small proteins. These translational fusions were cloned in a pET19b plasmid under T7 control. https://www.addgene.org/vector-database/2547/

| | |
|---|---|
| [L=299] (full-length notch) | MGLTPLMIAAVRGGGLDTGEDIENNEDSTAQVISDLLAQGAELNATMDKTGETSLHLAARFARADAAKRLLDAGADANSQDNTGRTPL HAAVAADAMGVFQILLRNRATNLNARMHDGTTPLILAARLAIEGMVEDLITADADINAADNSGKTALHWAAAVNNTEAVNILLMHHA NRDAQDDKDETPLFLAAREGSYEASKALLDNFANREITDHMDRLPRDVASERLHHDIVRLLDEHSGSGFAYGIKDPIYQKTLVPGQQNAT WIVPPGQYFMMGDWMSSFSTPVWISQAQGIRAGPGSSDKQEGEWPTGLRLSRIGGIH* |
| L=299 FLc | MGLTPLMIAAVRGGGLDTGEDIENNEDSTAQVISDLLAQGAELNATMDKTGETSLHLAARFARADAAKRLLDAGADANSQDNTGRTPL HAAVAADAMGVFQILLRNRATNLNARMHDGTTPLILAARLAIEGMVEDLITADADINAADNSGKTALHWAAAVNNTEAVNILLMHHA NRDAQDDKDETPLFLAAREGSYEASKALLDNFANREITDHMDRLPRDVASERLHHDIVRLLDEHSGSGFAYGIKDPIYQKTLVPGQQNAT WIVPPGQYFMMGDWMSSFSTPVWISQAQGIRAG**A**GSSDKQEGEWPTGLRLSRIGGIH* |
| L=299 Ac | MGLTPLMIAAVRGGGLDTGEDIENNEDSTAQVISDLLAQGAELNATMDKTGETSLHLAARFARADAAKRLLDAGADANSQDNTGRTPL HAAVAADAMGVFQILLRNRATNLNARMHDGTTPLILAARLAIEGMVEDLITADADINAADNSGKTALHWAAAVNNTEAVNILLMHHA NRDAQDDKDETPLFLAAREGSYEASKALLDNFANREITDHMDRLPRDVASERLHHDIVRLLDEHSGSGFAYGIKDPIYQKTLVPGQQNAT WIVPPGQYFMMGDWMSSFSTPVWISQAQGIRAG* |
| [L=294] | MGLTPLMIAAVRGGGLDTGEDIENNEDSTAQVISDLLAQGAELNATMDKTGETSLHLAARFARADAAKRLLDAGADANSQDNTGRTPL HAAVAADAMGVFQILLRNRATNLNARMHDGTTPLILAARLAIEGMVEDLITADADINAADNSGKTALHWAAAVNNTEAVNILLMHHA |

| | |
|---|---|
| (full-length notch) | NRDAQDDKDETPLFLAAREGSYEASKALLDNFANREITDHMDRLPRDVASERLHHDIVRLLDEHAYGIKDPIYQKTLVPGQQNATWIVPPGQYFMMGDWMSSFSTPVWISQAQGIRAGPGSSDKQEGEWPTGLRLSRIGGIH* |
| [L=289] (full-length notch) | MGLTPLMIAAVRGGGLDTGEDIENNEDSTAQVISDLLAQGAELNATMDKTGETSLHLAARFARADAAKRLLDAGADANSQDNTGRTPLHAAVAADAMGVFQILLRNRATNLNARMHDGTTPLILAARLAIEGMVEDLITADADINAADNSGKTALHWAAAVNNTEAVNILLMHHANRDAQDDKDETPLFLAAREGSYEASKALLDNFANREITDHMDRLPRDVASERLHHDIVRLLDEHDPIYQKTLVPGQQNATWIVPPGQYFMMGDWMSSFSTPVWISQAQGIRAGPGSSDKQEGE+WPTGLRLSRIGGIH* |
| [L=284] (full-length notch) | MGLTPLMIAAVRGGGLDTGEDIENNEDSTAQVISDLLAQGAELNATMDKTGETSLHLAARFARADAAKRLLDAGADANSQDNTGRTPLHAAVAADAMGVFQILLRNRATNLNARMHDGTTPLILAARLAIEGMVEDLITADADINAADNSGKTALHWAAAVNNTEAVNILLMHHANRDAQDDKDETPLFLAAREGSYEASKALLDNFANREITDHMDRLPRDVASERLHHDIVRLLDEHKTLVPGQQNATWIVPPGQYFMMGDWMSSFSTPVWISQAQGIRAGPGSSDKQEGEWPTGLRLSRIGGIH* |
| [L=279] (full-length notch)<br><br>[L=279]-A125E/A126F (full-length notch) | MGLTPLMIAAVRGGGLDTGEDIENNEDSTAQVISDLLAQGAELNATMDKTGETSLHLAARFARADAAKRLLDAGADANSQDNTGRTPLHAAVAADAMGVFQILLRNRATNLNARMHDGTTPLILAARLAIEGMVEDLITADADINAADNSGKTALHWAAAVNNTEAVNILLMHHANRDAQDDKDETPLFLAAREGSYEASKALLDNFANREITDHMDRLPRDVASERLHHDIVRLLDEHGQQNATWIVPPGQYFMMGDWMSSFSTPVWISQAQGIRAGPGSSDKQEGEWPTGLRLSRIGGIH*<br><br>MGLTPLMIAAVRGGGLDTGEDIENNEDSTAQVISDLLAQGAELNATMDKTGETSLHLAARFARADAAKRLLDAGADANSQDNTGRTPLHAAVAADAMGVFQILLRNRATNLNARMHDGTTPLIL**EF**RLAIEGMVEDLITADADINAADNSGKTALHWAAAVNNTEAVNILLMHHANRDAQDDKDETPLFLAAREGSYEASKALLDNFANREITDHMDRLPRDVASERLHHDIVRLLDEHGQQNATWIVPPGQYFMMGDWMSSFSTPVWISQAQGIRAGPGSSDKQEGEWPTGLRLSRIGGIH* |
| [L=274] (full-length notch) | MGLTPLMIAAVRGGGLDTGEDIENNEDSTAQVISDLLAQGAELNATMDKTGETSLHLAARFARADAAKRLLDAGADANSQDNTGRTPLHAAVAADAMGVFQILLRNRATNLNARMHDGTTPLILAARLAIEGMVEDLITADADINAADNSGKTALHWAAAVNNTEAVNILLMHHANRDAQDDKDETPLFLAAREGSYEASKALLDNFANREITDHMDRLPRDVASERLHHDIVRLLDEHTWIVPPGQYFMMGDWMSSFSTPVWISQAQGIRAGPGSSDKQEGEWPTGLRLSRIGGIH* |
| [L=269] (full-length notch) | MGLTPLMIAAVRGGGLDTGEDIENNEDSTAQVISDLLAQGAELNATMDKTGETSLHLAARFARADAAKRLLDAGADANSQDNTGRTPLHAAVAADAMGVFQILLRNRATNLNARMHDGTTPLILAARLAIEGMVEDLITADADINAADNSGKTALHWAAAVNNTEAVNILLMHHANRDAQDDKDETPLFLAAREGSYEASKALLDNFANREITDHMDRLPRDVASERLHHDIVRLLDEHPGQYFMMGDWMSSFSTPVWISQAQGIRAGPGSSDKQEGEWPTGLRLSRIGGIH* |
| [L=264] (full-length notch) | MGLTPLMIAAVRGGGLDTGEDIENNEDSTAQVISDLLAQGAELNATMDKTGETSLHLAARFARADAAKRLLDAGADANSQDNTGRTPLHAAVAADAMGVFQILLRNRATNLNARMHDGTTPLILAARLAIEGMVEDLITADADINAADNSGKTALHWAAAVNNTEAVNILLMHHANRDAQDDKDETPLFLAAREGSYEASKALLDNFANREITDHMDRLPRDVASERLHHDIVRLLDEHMMGDWMSSFSTPVWISQAQGIRAGPGSSDKQEGEWPTGLRLSRIGGIH* |
| [L=259] (notch Truncation) | MGLTPLMIAAVRGGGLDTGEDIENNEDSTAQVISDLLAQGAELNATMDKTGETSLHLAARFARADAAKRLLDAGADANSQDNTGRTPLHAAVAADAMGVFQILLRNRATNLNARMHDGTTPLILAARLAIEGMVEDLITADADINAADNSGKTALHWAAAVNNTEAVNILLMHHANRDAQDDKDETPLFLAAREGSYEASKALLDNFANREITDHMDRLPRDVASERLHHDIVRMMGDWMSSFSTPVWISQAQGIRAGPGSSDKQEGEWPTGLRLSRIGGIH* |
| [L=254] (notch Truncation) | MGLTPLMIAAVRGGGLDTGEDIENNEDSTAQVISDLLAQGAELNATMDKTGETSLHLAARFARADAAKRLLDAGADANSQDNTGRTPLHAAVAADAMGVFQILLRNRATNLNARMHDGTTPLILAARLAIEGMVEDLITADADINAADNSGKTALHWAAAVNNTEAVNILLMHHANRDAQDDKDETPLFLAAREGSYEASKALLDNFANREITDHMDRLPRDVASERLHMMGDWMSSFSTPVWISQAQGIRAGPGSSDKQEGEWPTGLRLSRIGGIH* |
| [L=244] (notch Truncation) | MGLTPLMIAAVRGGGLDTGEDIENNEDSTAQVISDLLAQGAELNATMDKTGETSLHLAARFARADAAKRLLDAGADANSQDNTGRTPLHAAVAADAMGVFQILLRNRATNLNARMHDGTTPLILAARLAIEGMVEDLITADADINAADNSGKTALHWAAAVNNTEAVNILLMHHANRDAQDDKDETPLFLAAREGSYEASKALLDNFANREITDHMDRLMMGDWMSSFSTPVWISQAQGIRAGPGSSDKQEGEWPTGLRLSRIGGIH* |
| [L=234] (notch Truncation) | MGLTPLMIAAVRGGGLDTGEDIENNEDSTAQVISDLLAQGAELNATMDKTGETSLHLAARFARADAAKRLLDAGADANSQDNTGRTPLHAAVAADAMGVFQILLRNRATNLNARMHDGTTPLILAARLAIEGMVEDLITADADINAADNSGKTALHWAAAVNNTEAVNILLMHHANRDAQDDKDETPLFLAAREGSYEASKALLDNFANMMGDWMSSFSTPVWISQAQGIRAGPGSSDKQEGEWPTGLRLSRIGGIH* |
| [L=229] (notch Truncation) | MGLTPLMIAAVRGGGLDTGEDIENNEDSTAQVISDLLAQGAELNATMDKTGETSLHLAARFARADAAKRLLDAGADANSQDNTGRTPLHAAVAADAMGVFQILLRNRATNLNARMHDGTTPLILAARLAIEGMVEDLITADADINAADNSGKTALHWAAAVNNTEAVNILLMHHANRDAQDDKDETPLFLAAREGSYEASKALLMMGDWMSSFSTPVWISQAQGIRAGPGSSDKQEGEWPTGLRLSRIGGIH* |
| [L=224] (notch Truncation) | MGLTPLMIAAVRGGGLDTGEDIENNEDSTAQVISDLLAQGAELNATMDKTGETSLHLAARFARADAAKRLLDAGADANSQDNTGRTPLHAAVAADAMGVFQILLRNRATNLNARMHDGTTPLILAARLAIEGMVEDLITADADINAADNSGKTALHWAAAVNNTEAVNILLMHHANRDAQDDKDETPLFLAAREGSYEAMMGDWMSSFSTPVWISQAQGIRAGPGSSDKQEGEWPTGLRLSRIGGIH* |

| | |
|---|---|
| [L=214] (notch Truncation) | MGLTPLMIAAVRGGGLDTGEDIENNEDSTAQVISDLLAQGAELNATMDKTGETSLHLAARFARADAAKRLLDAGADANSQDNTGRTPLHAAVAADAMGVFQILLRNRATNLNARMHDGTTPLILAARLAIEGMVEDLITADADINAADNSGKTALHWAAAVNNTEAVNILLMHHANRDAQDDKDETPLFMMGDWMSSFSTPVWISQAQGIRAGPGSSDKQEGEWPTGLRLSRIGGIH* |
| [L=209] (notch Truncation) | MGLTPLMIAAVRGGGLDTGEDIENNEDSTAQVISDLLAQGAELNATMDKTGETSLHLAARFARADAAKRLLDAGADANSQDNTGRTPLHAAVAADAMGVFQILLRNRATNLNARMHDGTTPLILAARLAIEGMVEDLITADADINAADNSGKTALHWAAAVNNTEAVNILLMHHANRDAQDDKDMMGDWMSSFSTPVWISQAQGIRAGPGSSDKQEGEWPTGLRLSRIGGIH* |
| [L=204] (notch Truncation) | MGLTPLMIAAVRGGGLDTGEDIENNEDSTAQVISDLLAQGAELNATMDKTGETSLHLAARFARADAAKRLLDAGADANSQDNTGRTPLHAAVAADAMGVFQILLRNRATNLNARMHDGTTPLILAARLAIEGMVEDLITADADINAADNSGKTALHWAAAVNNTEAVNILLMHHANRDAMMGDWMSSFSTPVWISQAQGIRAGPGSSDKQEGEWPTGLRLSRIGGIH* |
| [L=199] (notch Truncation) | MGLTPLMIAAVRGGGLDTGEDIENNEDSTAQVISDLLAQGAELNATMDKTGETSLHLAARFARADAAKRLLDAGADANSQDNTGRTPLHAAVAADAMGVFQILLRNRATNLNARMHDGTTPLILAARLAIEGMVEDLITADADINAADNSGKTALHWAAAVNNTEAVNILLMHHMMGDWMSSFSTPVWISQAQGIRAGPGSSDKQEGEWPTGLRLSRIGGIH* |
| [L=194] (notch Truncation) | MGLTPLMIAAVRGGGLDTGEDIENNEDSTAQVISDLLAQGAELNATMDKTGETSLHLAARFARADAAKRLLDAGADANSQDNTGRTPLHAAVAADAMGVFQILLRNRATNLNARMHDGTTPLILAARLAIEGMVEDLITADADINAADNSGKTALHWAAAVNNTEAVNIMMGDWMSSFSTPVWISQAQGIRAGPGSSDKQEGEWPTGLRLSRIGGIH* |
| [L=189] (notch Truncation) | MGLTPLMIAAVRGGGLDTGEDIENNEDSTAQVISDLLAQGAELNATMDKTGETSLHLAARFARADAAKRLLDAGADANSQDNTGRTPLHAAVAADAMGVFQILLRNRATNLNARMHDGTTPLILAARLAIEGMVEDLITADADINAADNSGKTALHWAAAVNNTMMGDWMSSFSTPVWISQAQGIRAGPGSSDKQEGEWPTGLRLSRIGGIH* |
| [L=184] (notch Truncation) | MGLTPLMIAAVRGGGLDTGEDIENNEDSTAQVISDLLAQGAELNATMDKTGETSLHLAARFARADAAKRLLDAGADANSQDNTGRTPLHAAVAADAMGVFQILLRNRATNLNARMHDGTTPLILAARLAIEGMVEDLITADADINAADNSGKTALHWAAMMGDWMSSFSTPVWISQAQGIRAGPGSSDKQEGEWPTGLRLSRIGGIH* |
| [L=179] (notch Truncation) | MGLTPLMIAAVRGGGLDTGEDIENNEDSTAQVISDLLAQGAELNATMDKTGETSLHLAARFARADAAKRLLDAGADANSQDNTGRTPLHAAVAADAMGVFQILLRNRATNLNARMHDGTTPLILAARLAIEGMVEDLITADADINAADNSGKTAMMGDWMSSFSTPVWISQAQGIRAGPGSSDKQEGEWPTGLRLSRIGGIH* |
| [L=174] (notch Truncation) | MGLTPLMIAAVRGGGLDTGEDIENNEDSTAQVISDLLAQGAELNATMDKTGETSLHLAARFARADAAKRLLDAGADANSQDNTGRTPLHAAVAADAMGVFQILLRNRATNLNARMHDGTTPLILAARLAIEGMVEDLITADADINAADNMMGDWMSSFSTPVWISQAQGIRAGPGSSDKQEGEWPTGLRLSRIGGIH* |
| [L=169] (notch Truncation) | MGLTPLMIAAVRGGGLDTGEDIENNEDSTAQVISDLLAQGAELNATMDKTGETSLHLAARFARADAAKRLLDAGADANSQDNTGRTPLHAAVAADAMGVFQILLRNRATNLNARMHDGTTPLILAARLAIEGMVEDLITADADIMMGDWMSSFSTPVWISQAQGIRAGPGSSDKQEGEWPTGLRLSRIGGIH* |
| [L=164] (notch Truncation) | MGLTPLMIAAVRGGGLDTGEDIENNEDSTAQVISDLLAQGAELNATMDKTGETSLHLAARFARADAAKRLLDAGADANSQDNTGRTPLHAAVAADAMGVFQILLRNRATNLNARMHDGTTPLILAARLAIEGMVEDLITMMGDWMSSFSTPVWISQAQGIRAGPGSSDKQEGEWPTGLRLSRIGGIH* |
| [L=159] (notch Truncation) | MGLTPLMIAAVRGGGLDTGEDIENNEDSTAQVISDLLAQGAELNATMDKTGETSLHLAARFARADAAKRLLDAGADANSQDNTGRTPLHAAVAADAMGVFQILLRNRATNLNARMHDGTTPLILAARLAIEGMVMMGDWMSSFSTPVWISQAQGIRAGPGSSDKQEGEWPTGLRLSRIGGIH* |
| [L=154] (notch Truncation) | MGLTPLMIAAVRGGGLDTGEDIENNEDSTAQVISDLLAQGAELNATMDKTGETSLHLAARFARADAAKRLLDAGADANSQDNTGRTPLHAAVAADAMGVFQILLRNRATNLNARMHDGTTPLILAARLAMMGDWMSSFSTPVWISQAQGIRAGPGSSDKQEGEWPTGLRLSRIGGIH* |
| [L=149] (notch Truncation) | MGLTPLMIAAVRGGGLDTGEDIENNEDSTAQVISDLLAQGAELNATMDKTGETSLHLAARFARADAAKRLLDAGADANSQDNTGRTPLHAAVAADAMGVFQILLRNRATNLNARMHDGTTPLILMMGDWMSSFSTPVWISQAQGIRAGPGSSDKQEGEWPTGLRLSRIGGIH* |
| [L=144] (notch Truncation) | MGLTPLMIAAVRGGGLDTGEDIENNEDSTAQVISDLLAQGAELNATMDKTGETSLHLAARFARADAAKRLLDAGADANSQDNTGRTPLHAAVAADAMGVFQILLRNRATNLNARMHDGTMMGDWMSSFSTPVWISQAQGIRAGPGSSDKQEGEWPTGLRLSRIGGIH* |
| [L=139] (notch Truncation) | MGLTPLMIAAVRGGGLDTGEDIENNEDSTAQVISDLLAQGAELNATMDKTGETSLHLAARFARADAAKRLLDAGADANSQDNTGRTPLHAAVAADAMGVFQILLRNRATNLNARMMGDWMSSFSTPVWISQAQGIRAGPGSSDKQEGEWPTGLRLSRIGGIH* |
| [L=134] (notch Truncation) | MGLTPLMIAAVRGGGLDTGEDIENNEDSTAQVISDLLAQGAELNATMDKTGETSLHLAARFARADAAKRLLDAGADANSQDNTGRTPLHAAVAADAMGVFQILLRNRATMMGDWMSSFSTPVWISQAQGIRAGPGSSDKQEGEWPTGLRLSRIGGIH* |
| [L=129] | MGLTPLMIAAVRGGGLDTGEDIENNEDSTAQVISDLLAQGAELNATMDKTGETSLHLAARFARADAAKRLLDAGADANSQDNTGRTPLHAAVAADAMGVFQILLMMGDWMSSFSTPVWISQAQGIRAGPGSSDKQEGEWPTGLRLSRIGGIH* |

| | |
|---|---|
| (notch Truncation) | |
| [L=124] (notch Truncation) | MGLTPLMIAAVRGGGLDTGEDIENNEDSTAQVISDLLAQGAELNATMDKTGETSLHLAARFARADAAKRLLDAGADANSQDNTGRTPL<br>HAAVAADAMGVMMGDWMSSFSTPVWISQAQGIRAGPGSSDKQEGEWPTGLRLSRIGGIH* |
| [L=119] (notch Truncation) | MGLTPLMIAAVRGGGLDTGEDIENNEDSTAQVISDLLAQGAELNATMDKTGETSLHLAARFARADAAKRLLDAGADANSQDNTGRTPL<br>HAAVAAMMGDWMSSFSTPVWISQAQGIRAGPGSSDKQEGEWPTGLRLSRIGGIH* |
| [L=114] (notch Truncation) | MGLTPLMIAAVRGGGLDTGEDIENNEDSTAQVISDLLAQGAELNATMDKTGETSLHLAARFARADAAKRLLDAGADANSQDNTGRTPL<br>HMMGDWMSSFSTPVWISQAQGIRAGPGSSDKQEGEWPTGLRLSRIGGIH* |
| [L=109] (notch Truncation) | MGLTPLMIAAVRGGGLDTGEDIENNEDSTAQVISDLLAQGAELNATMDKTGETSLHLAARFARADAAKRLLDAGADANSQDNTGMM<br>GDWMSSFSTPVWISQAQGIRAGPGSSDKQEGEWPTGLRLSRIGGIH* |
| [L=107] (notch Truncation) | MGLTPLMIAAVRGGGLDTGEDIENNEDSTAQVISDLLAQGAELNATMDKTGETSLHLAARFARADAAKRLLDAGADANSQDNMMGD<br>WMSSFSTPVWISQAQGIRAGPGSSDKQEGEWPTGLRLSRIGGIH* |
| [L=89] (notch Truncation) | MANRSFIYEPFQIPSGSMMPTLNSTDFILVEKFAYGIKDPIYQKTLIETGHPKRGDIVVFKYPEDPKLDYIKRAVGLPGDKVTYDPVSKELTIQ<br>PGCSSGQACENALPVTYSNVEPSDFVQTFSRRNGGEATSGFFEVPKQETKENGIRLSETSGSGSGLTPLMIAAVRGGGLDTGEDIENNED<br>STAQVISDLLAQGAELNATMDKTGETSLHLAARFARAMMGDWMSSFSTPVWISQAQGIRAGPGSSDKQEGEWPTGLRLSRIGGIH* |
| [L=84] (notch Truncation) | MANRSFIYEPFQIPSGSMMPTLNSTDFILVEKFAYGIKDPIYQKTLIETGHPKRGDIVVFKYPEDPKLDYIKRAVGLPGDKVTYDPVSKELTIQ<br>PGCSSGQACENALPVTYSNVEPSDFVQTFSRRNGGEATSGFFEVPKQETKENGIRLSETSGSGSGLTPLMIAAVRGGGLDTGEDIENNED<br>STAQVISDLLAQGAELNATMDKTGETSLHLAAMMGDWMSSFSTPVWISQAQGIRAGPGSSDKQEGEWPTGLRLSRIGGIH* |
| [L=79] (notch Truncation) | MANRSFIYEPFQIPSGSMMPTLNSTDFILVEKFAYGIKDPIYQKTLIETGHPKRGDIVVFKYPEDPKLDYIKRAVGLPGDKVTYDPVSKELTIQ<br>PGCSSGQACENALPVTYSNVEPSDFVQTFSRRNGGEATSGFFEVPKQETKENGIRLSETSGSGSGLTPLMIAAVRGGGLDTGEDIENNED<br>STAQVISDLLAQGAELNATMDKTGETSMMGDWMSSFSTPVWISQAQGIRAGPGSSDKQEGEWPTGLRLSRIGGIH* |
| [L=74] (notch Truncation) | MANRSFIYEPFQIPSGSMMPTLNSTDFILVEKFAYGIKDPIYQKTLIETGHPKRGDIVVFKYPEDPKLDYIKRAVGLPGDKVTYDPVSKELTIQ<br>PGCSSGQACENALPVTYSNVEPSDFVQTFSRRNGGEATSGFFEVPKQETKENGIRLSETSGSGSGLTPLMIAAVRGGGLDTGEDIENNED<br>STAQVISDLLAQGAELNATMDKMMGDWMSSFSTPVWISQAQGIRAGPGSSDKQEGEWPTGLRLSRIGGIH* |
| [L=69] (notch Truncation) | MANRSFIYEPFQIPSGSMMPTLNSTDFILVEKFAYGIKDPIYQKTLIETGHPKRGDIVVFKYPEDPKLDYIKRAVGLPGDKVTYDPVSKELTIQ<br>PGCSSGQACENALPVTYSNVEPSDFVQTFSRRNGGEATSGFFEVPKQETKENGIRLSETSGSGSGLTPLMIAAVRGGGLDTGEDIENNED<br>STAQVISDLLAQGAELNMMGDWMSSFSTPVWISQAQGIRAGPGSSDKQEGEWPTGLRLSRIGGIH* |
| [L=64] (notch Truncation) | MANRSFIYEPFQIPSGSMMPTLNSTDFILVEKFAYGIKDPIYQKTLIETGHPKRGDIVVFKYPEDPKLDYIKRAVGLPGDKVTYDPVSKELTIQ<br>PGCSSGQACENALPVTYSNVEPSDFVQTFSRRNGGEATSGFFEVPKQETKENGIRLSETSGSGSGLTPLMIAAVRGGGLDTGEDIENNED<br>STAQVISDLLAQMMGDWMSSFSTPVWISQAQGIRAGPGSSDKQEGEWPTGLRLSRIGGIH* |
| [L=59] (notch Truncation) | MANRSFIYEPFQIPSGSMMPTLNSTDFILVEKFAYGIKDPIYQKTLIETGHPKRGDIVVFKYPEDPKLDYIKRAVGLPGDKVTYDPVSKELTIQ<br>PGCSSGQACENALPVTYSNVEPSDFVQTFSRRNGGEATSGFFEVPKQETKENGIRLSETSGSGSGLTPLMIAAVRGGGLDTGEDIENNED<br>STAQVISMMGDWMSSFSTPVWISQAQGIRAGPGSSDKQEGEWPTGLRLSRIGGIH* |
| [L=45] (notch Truncation) | MANRSFIYEPFQIPSGSMMPTLNSTDFILVEKFAYGIKDPIYQKTLIETGHPKRGDIVVFKYPEDPKLDYIKRAVGLPGDKVTYDPVSKELTIQ<br>PGCSSGQACENALPVTYSNVEPSDFVQTFSRRNGGEATSGFFEVPKQETKENGIRLSETSGSGSGLTPLMIAAVRGGGLDTGEMMGDW<br>MSSFSTPVWISQAQGIRAGPGSSDKQEGEWPTGLRLSRIGGIH* |
| [L=43] (notch Truncation) | MANRSFIYEPFQIPSGSMMPTLNSTDFILVEKFAYGIKDPIYQKTLIETGHPKRGDIVVFKYPEDPKLDYIKRAVGLPGDKVTYDPVSKELTIQ<br>PGCSSGQACENALPVTYSNVEPSDFVQTFSRRNGGEATSGFFEVPKQETKENGIRLSETSGSGSGLTPLMIAAVRGGGLDTMMGDWMS<br>SFSTPVWISQAQGIRAGPGSSDKQEGEWPTGLRLSRIGGIH* |
| [L=41] (notch Truncation) | MANRSFIYEPFQIPSGSMMPTLNSTDFILVEKFAYGIKDPIYQKTLIETGHPKRGDIVVFKYPEDPKLDYIKRAVGLPGDKVTYDPVSKELTIQ<br>PGCSSGQACENALPVTYSNVEPSDFVQTFSRRNGGEATSGFFEVPKQETKENGIRLSETSGSGSGLTPLMIAAVRGGMMGDWMSSFST<br>PVWISQAQGIRAGPGSSDKQEGEWPTGLRLSRIGGIH* |

**Supplementary figure 3, exemplary SDS/PAGE gels for various constructs.**

To make an initial identification of the arrested and full-length bands we created arrested (P17 to stop codon mutation in arrest peptide SecM *E. coli*) and full-length (P17A inactivating mutation in arrest peptide SecM *E. coli*) controls. In general, the two bands to be integrated are the first and the second. The rest of the bands are believed to be random truncated versions created by ribosomal stacking (Simms, Yan et al. 2017). This phenomenon is more prominent with repeat proteins.

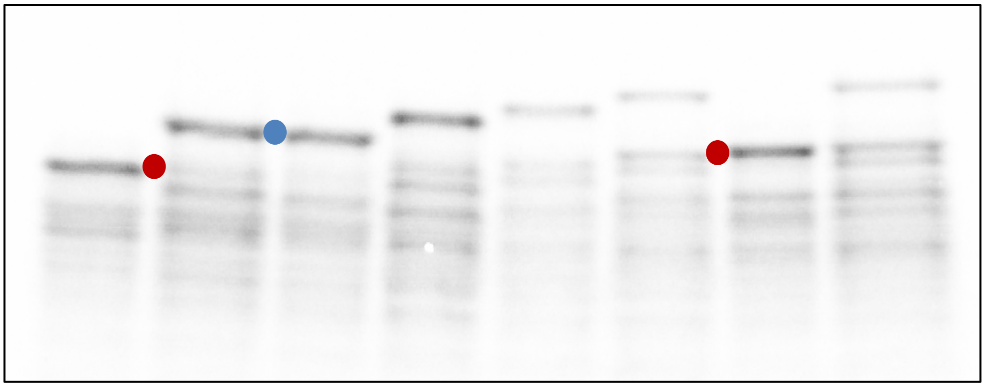

Gel 1. Lanes= 1) L279 Arrested control, 2) L279, 3) L279 Full length control, 4) L284, 5) L289, 6) L294, 7) L299-Arrested control, 8) L299.

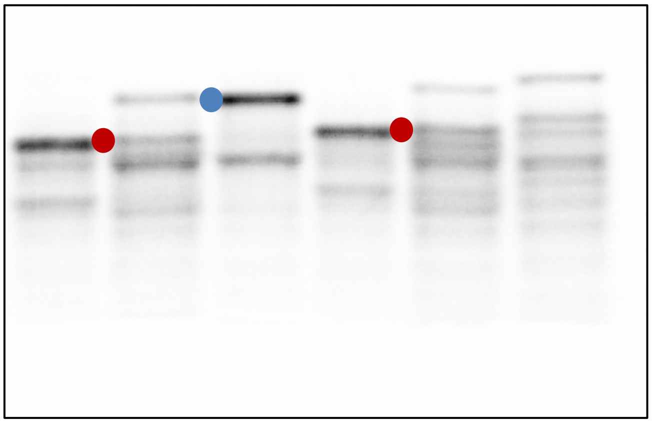

Gel 2. Lanes= 1) L264 Arrested control, 2) L264, 3) L264 Full length control, 4) L269 Arrested control, 5) L269, 6) L274

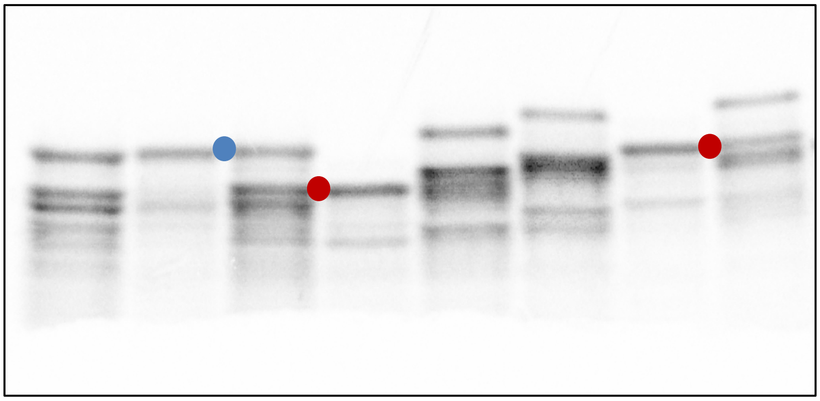

Gel 3. Lanes= 1) L229, 2) L234 Full length control, 3) L234, 4) L234 Arrested control, 5) L244, 6) L254, 7) L259 Arrested control, 8) L259.

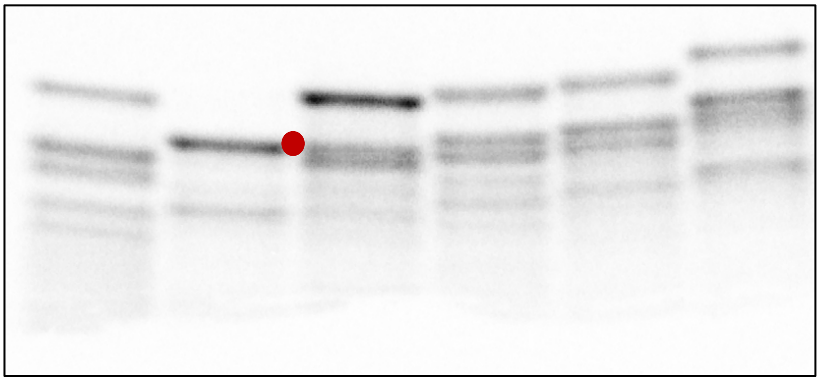

Gel 4. Lanes= 1) L214, 2) L224 Arrested control, 3) L224, 4) L229, 5) L234, 6) L244

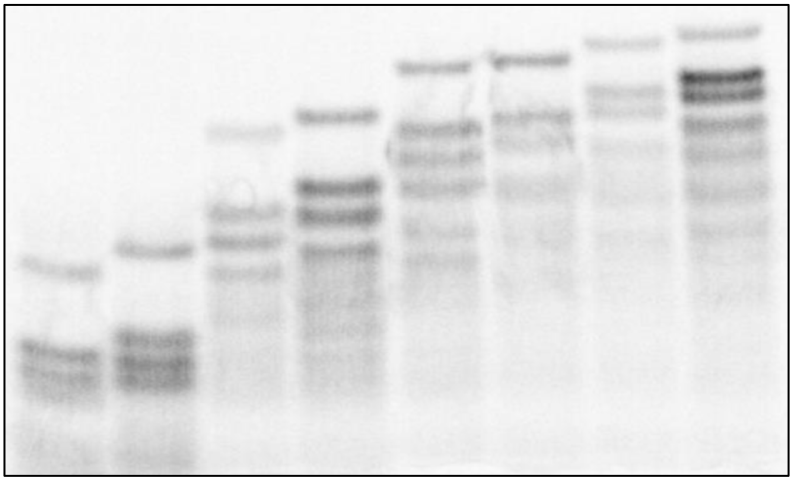

Gel 5. Lanes= 1) L114, 2) L124, 3) L159, 4) L164, 5) L179, 6) L184, 7) L194, 8) L199

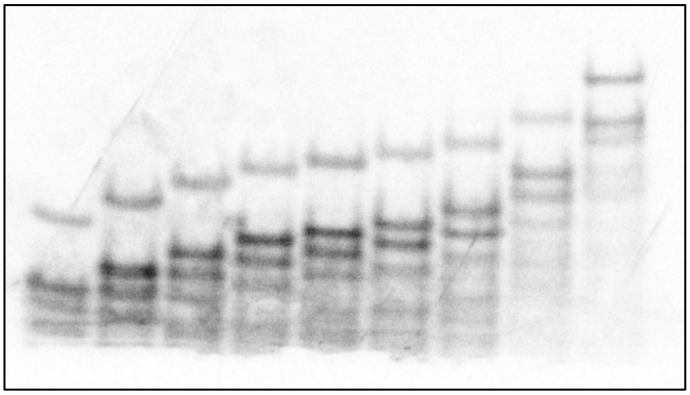

Gel 6. Lanes= 1) L119, 2) L129, 3) L134, 4) L139, 5) L144, 6) L149, 7) L154, 8) L169, 9) L189

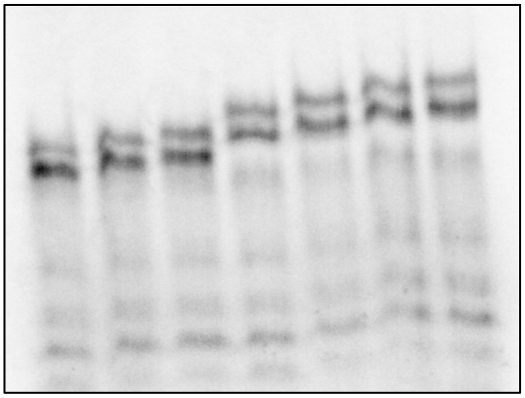

Gel 7. Lanes= 1) L41, 2) L43, 3) L45, 4) L59, 5) L64, 6) L69, 7) L75

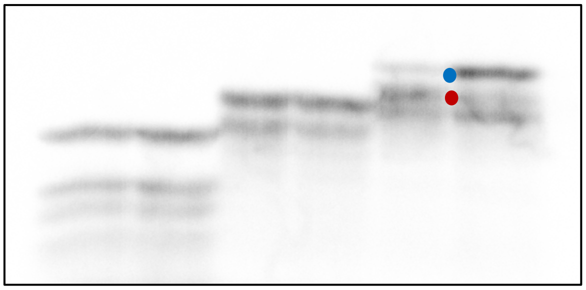

Gel 8. Lanes= 1) L189(A125E/A126F), 2) L189, 3) L224(A125E/A126F), 4) L224, 5) L279 (A125E/A126F), 6) L279.

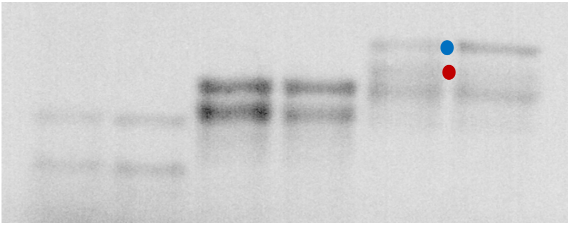

Gel 9. Lanes= 1) L189(A125E/A126F), 2) L189, 3) L224(A125E/A126F), 4) L224, 5) L279 (A125E/A126F), 6) L279.

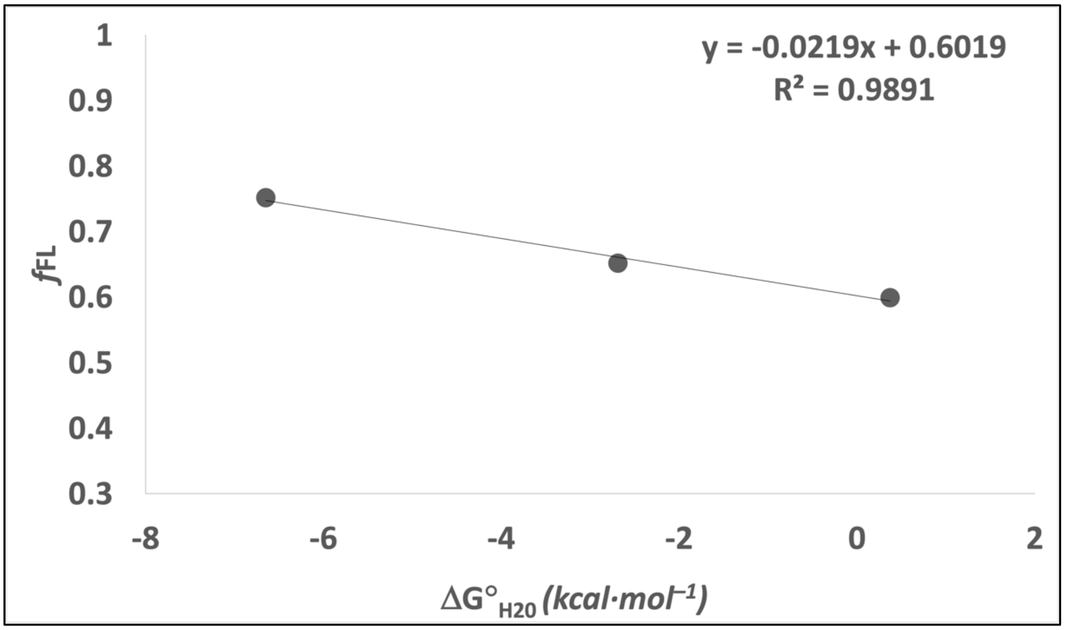


**Supplementary figure 4**. Linear fit of measured $f_{FL}$ *values* of the three peaks discussed in the text and the corresponding experimentally determined ΔG° values in (Mello and Barrick 2004).

| **Construct** | **DG°$_{H20}$** | **M2 DG°$_{H20}$** | ***$f_{FL}$*** | **M2 *$f_{FL}$*** |
|---|---|---|---|---|
| Nank1—7/L279 | -6.65±0.04 | ≅ 0.1 | 0.75±0.015 | 0.37±0.04 |
| Nank1—5/L224 | -2.69±0.04 | ND | 0.65±0.041 | 0.62±0.10 |
| Nank1—4/L189 | +0.37 | ND | 0.59±0.054 | 0.53±0.09 |

DG°$_{H20}$ *(kcal·mol–1)*
$f_{FL} = I_{FL} / (I_{FL} + I_A)$
**M2**(A119E and A120F), **ND** (No disponible)

**Supplementary table 2**. Experimentally determined ΔG° for indicated constructs (taken from (Mello and Barrick 2004)) and its corresponding $f_{FL}$ measured in the present work. The M2 is a mutation (A125E and A126F) that decreases the ΔG° of the full-length construct close to 0. The ΔG° have not been determined for the mutated-truncated versions of the Notch Ankyrin protein. Measured levels of these mutations by $f_{FL}$ (standard deviations are shown).

**Supplementary Figure 5. Integration procedure.** After drying the SDS-PAGE GEL, they are used to expose 16 Hrs. a BAS-IP MS 2025 E film. The scanning procedure in a Phosphoimager produces the tiff files used for integration.

Vertical plot with ImageJ

https://imagej.nih.gov/ij/download.html

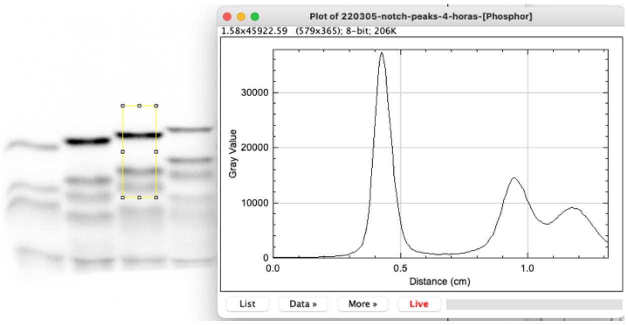


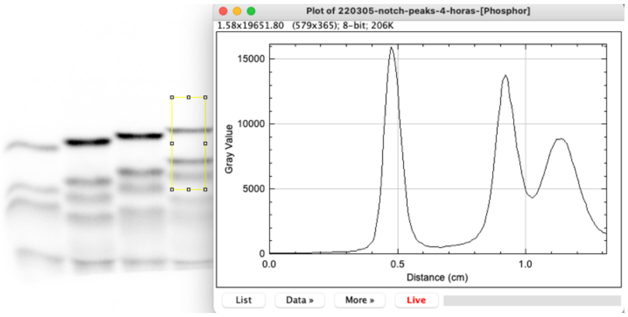


Integration using in house software

Easy Quant (available upon request)

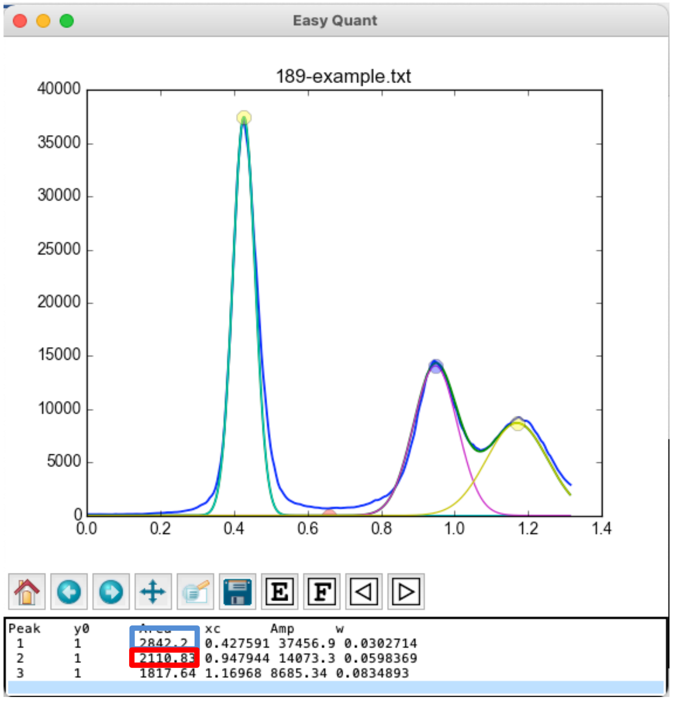


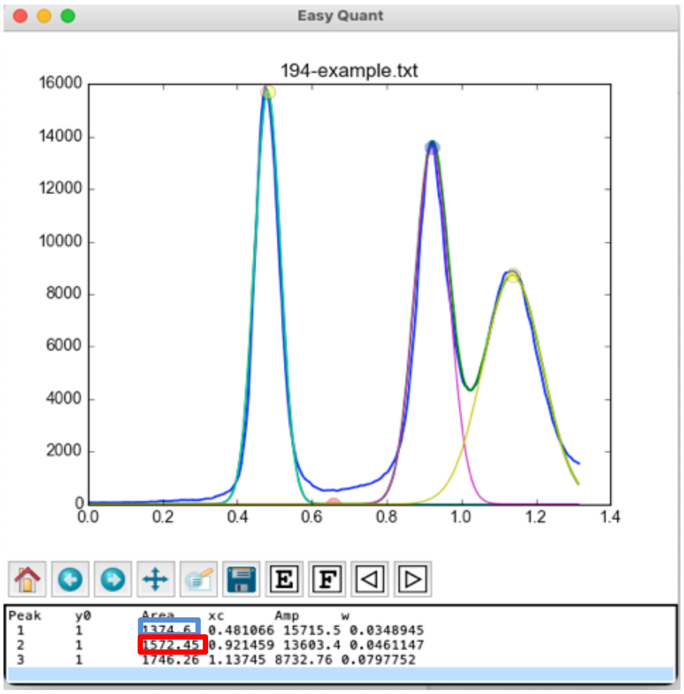

$$f_{FL} = I_{FL} / I_{FL} + I_A$$

Plot $f_{FL}$ for each construct

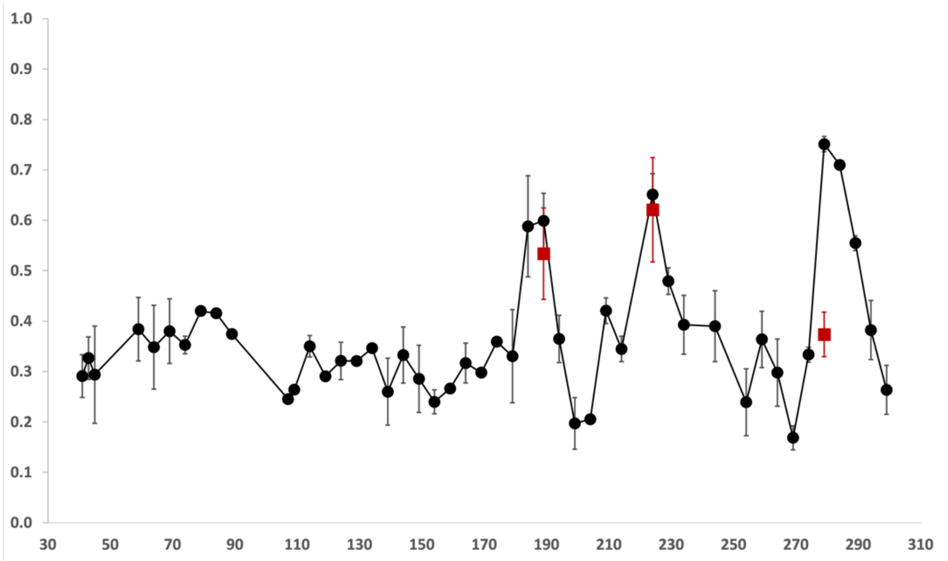


**Supplementary references**


Mello, C. C. and D. Barrick (2004). "An experimentally determined protein folding energy landscape." Proc Natl Acad Sci U S A **101**(39): 14102-14107.

Simms, C. L., L. L. Yan and H. S. Zaher (2017). "Ribosome Collision Is Critical for Quality Control during No-Go Decay." Mol Cell **68**(2): 361-373 e365.
